# Functions of enzyme domains in 2-methylisoborneol biosynthesis and enzymatic synthesis of non-natural analogs

**DOI:** 10.3762/bjoc.19.104

**Published:** 2023-09-22

**Authors:** Binbin Gu, Lin-Fu Liang, Jeroen S Dickschat

**Affiliations:** 1 Kekulé-Institute of Organic Chemistry and Biochemistry, University of Bonn, Gerhard-Domagk-Straße 1, 53121 Bonn, Germany

**Keywords:** biosynthesis, enzymes, isotopes, substrate analogs, terpenes

## Abstract

Two aspects of the biosynthesis of the non-canonical terpene synthase for 2-methylisoborneol have been studied. Several 2-methylisoborneol synthases have a proline-rich N-terminal domain of unknown function. The results presented here demonstrate that this domain leads to a reduced enzyme activity, in addition to its ability to increase long-term solubility of the protein. Furthermore, the substrate scope of the 2-methylisoborneol synthase was investigated through enzyme incubations with several substrate analogs, giving access to two C_12_ monoterpenoids. Implications on the stereochemical course of the terpene cyclisation by 2-methylisoborneol synthase are discussed.

## Introduction

**Scheme 1:**
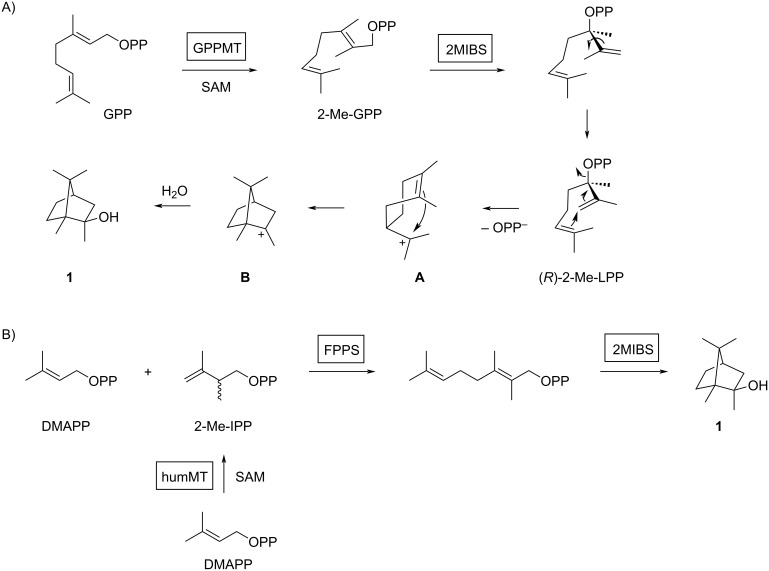
The biosynthesis of 2-methylisoborneol (**1**). A) SAM-dependent methylation of GPP to 2-Me-GPP by GPPMT and terpene cyclisation to **1** by 2MIBS. B) Non-natural formation using the enzymes humMT for the methylation of DMAPP to 2-Me-IPP, FPPS for the coupling of DMAPP and 2-Me-IPP to 2-Me-GPP, and 2MIBS for the conversion into **1**.

The musty odorant 2-methylisoborneol (**1**, Scheme 1) has first been obtained through synthesis from camphor [1] and has subsequently been discovered as a natural product in streptomycetes [2–3]. The volatile compound was later also found in various other bacterial lineages including actinobacteria [4–9], myxobacteria [10] and aquatic cyanobacteria [11–12], as well as in liverwort [13] and ascomycete fungi [14]. The odour qualities of **1** may be concentration dependent and range from musty at low concentrations (<1 μg L^−1^) to camphoraceous at higher concentrations (10 μg L^−1^) [15]. The compound has received considerable attention because of its potential to cause odour episodes in water supply systems [16–17]. The production of **1** by *Penicillium* can add to the flavour of cheese [18], while its occurrence in fish and coffee leads to an unpleasant off-flavour [19–20].

The biosynthesis of **1** has been extensively studied. The initial hypothesis that **1** could be a degraded sesquiterpene [3] was not confirmed through isotopic labelling experiments that rather pointed to a methylated monoterpene [10,21]. Based on these experiments a biosynthetic model was proposed that proceeds through the *S*-adenosylmethionine (SAM) dependent methylation of geranyl diphosphate (GPP) to 2-methyl-GPP (2-Me-GPP), followed by a terpene cyclisation to **1** (Scheme 1A) [10]. The cyclisation cascade requires isomerisation to (*R*)-2-methyllinalyl diphosphate [22], followed by two sequential cyclisation reactions to **A** and **B**, and terminal quenching with water. This hypothesis was confirmed by the discovery of the biosynthetic genes coding for a GPP methyltransferase (GPPMT) and a type I terpene synthase termed 2-methylisoborneol synthase (2MIBS) [23–24]. Interestingly, the pathway to **1** can be reconstituted in vitro using the methyltransferase humMT from *Micromonospora humi* for the methylation of dimethylallyl diphosphate (DMAPP) to 2-methylisopentenyl diphosphate (2-Me-IPP) [25], followed by coupling with DMAPP to 2-Me-GPP and terpene cyclisation using farnesyl diphosphate synthase (FPPS) and 2MIBS from *Streptomyces coelicolor* [26] (Scheme 1B).

Crystal structures of both enzymes have been obtained [27–28] and allowed for a deep structure-based investigation of 2MIBS through site-directed mutagenesis [29]. The predicted amino acid sequences of 2MIBS homologs from different organisms can have a variable lengths ranging from ca. 330 amino acids (e.g., in *Longispora albida* DSM 44784, accession number WP_018349754, 329 amino acids) to more than 550 amino acids (e.g., in *Nocardia amikacinitolerans* DSM 45535, accession number WP_253814817, 580 amino acids). The long versions of 2MIBSs exhibit a proline-rich N-terminal domain of unknown function that appears disordered in the crystal structure [28]. As a first aspect of this study, we have investigated the possible function of this N-terminal domain.

2MIBS is known to form several methylated monoterpenes as side products that have been identified by GC–MS analysis and the synthesis of reference compounds [30–31]. Notably, 2MIBS also shows some substrate flexibility and can convert GPP into monoterpenes in vitro, albeit with less efficiency as compared to the conversion of 2-Me-GPP [24]. On the other hand, single residue switches also allow for the acceptance of 2-Me-GPP and conversion into C_11_ compounds by plant terpene synthases during heterologous expression in yeast [32]. These findings prompted us to investigate in a second aspect of this study whether 2MIBS is able to convert non-natural GPP analogs with changed alkylation pattern.

## Results and Discussion

### Function of the proline-rich N-terminal domain of 2MIBS

*S. coelicolor* 2MIBS was selected to investigate the function of the proline-rich N-terminal domain (hereafter termed A domain, the C-terminal domain is named as domain B). Based on a sequence alignment with the short 2MIBS from *Longispora albida* DSM 44784 (WP_018349754) the border between domains A and B in the 2MIBS from *S. coelicolor* was identified. Domain A spans the amino acid residues 1–115 and domain B includes the amino acid residues 115–440. The gene sequences for both domains were cloned individually, heterologously expressed as N-terminally His-tagged proteins in *Escherichia coli* and purified (Supporting Information File 1, Figure S1). Also full length 2MIBS from *S. coelicolor* was obtained in the same way.

**Figure 1:**
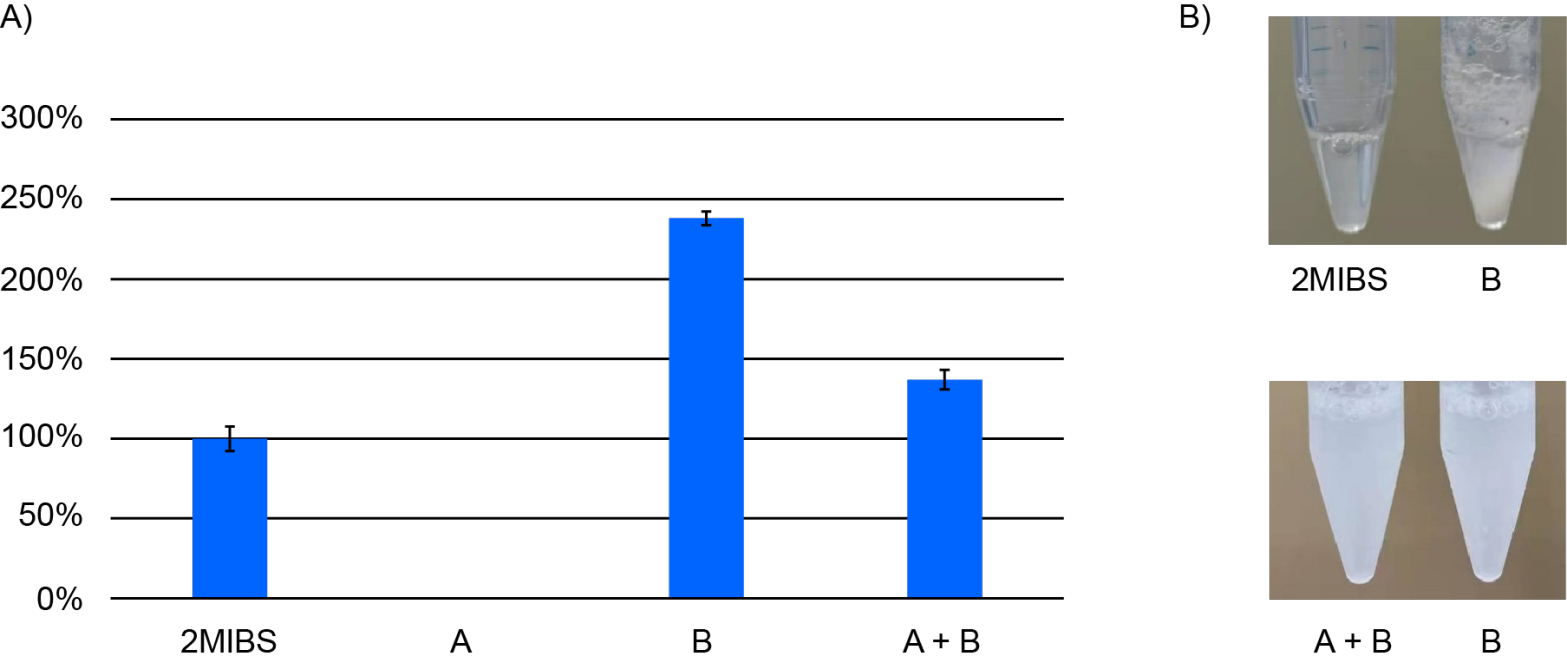
The function of the N-terminal A domain of 2MIBS. A) Relative production of **1** with full length 2MIBS from *S. coelicolor* (normalised to 100%), domain A, domain B, and domains A + B. The bars indicate mean and standard deviation from triplicates. B) Enzyme precipitation after 12 h in elution buffer at 4 °C.

The relative production of **1** from 2-Me-GPP was tested in triplicate reactions with full length 2MIBS, domain A, domain B, and the combination of domains A + B (Figure 1A). Herein, the production of full length 2MIBS was normalised to 100%. Domain A alone did not yield any enzyme product, whereas domain B alone gave a strongly increased production of **1** (238 ± 4%). The combination of domains A and B resulted in a moderately enhanced production of **1** (137 ± 6%). In other words, domain A, especially if it is covalently bound to domain B, serves as a gatekeeper that limits the production of **1** by 2MIBS. Furthermore, a difference was observed in the long-term solubility in the elution buffer used for protein purification through Ni^2+^-NTA affinity chromatography. While domain B alone showed a substantial enzyme precipitation after 12 h at 4 °C, full length 2MIBS did not (Figure 1B), suggesting that the A domain increases enzyme solubility and long-term stability. This effect needs a covalent bond between the two domains A and B, as indicated by a similar precipitation of domain B alone and domain B in the presence of domain A. In these experiments, the N-terminal His-tags at both domains A and B may influence protein–protein interaction with the consequence that the mixture of the individually expressed domains shows a similarly rapid precipitation as domain B alone. However, some interaction between the individually expressed domains A and B can be concluded from their reduced productivity in comparison to domain B alone.

### Enzymatic synthesis of non-natural analogs of 2-methylisoborneol

For the enzymatic preparation of non-natural analogs of **1** different combinations of DMAPP and IPP derivatives were used (Table 1). The synthesis of the DMAPP analogs **DA-1**, **DA-2** and **DA-6** and of the IPP analogs **IA-1**, **IA-2** and **IA-3** was reported previously [26,33–34], and **DA-3**, **DA-4** and **DA-5** were obtained through standard phosphorylation of the corresponding known alcohols [35–37]. In a first screening, these derivatives were tested in all possible combinations in small scale reactions (0.3 mg of each substrate) with FPPS. The results were investigated through dephosphorylation with calf intestinal phosphatase (CIP), followed by extraction of the reaction mixture with hexane and GC–MS analysis. Several combinations of DMAPP and IPP analogs resulted in a good production of the corresponding GPP analogs (indicated by three black plus signs in Table 1). Other combinations gave a medium (++) or low production (+), while some of the substrate combinations were unsuccessful. For the successful cases a second screening was performed by small scale incubations of DMAPP and IPP analogs with FPPS and 2MIBS. Besides the combination of DMAPP and IPP leading to GPP, a known poor substrate of 2MIBS [24], and the combination of DMAPP and **IA-1** that was previously reported to yield the natural substrate of 2MIBS 2-Me-GPP [26], only two substrate combinations (**DA-4** + **IA-1** and **DA-5** + **IA-1**) gave access to analogs of **1**. The production of terpenoids by FPPS and 2MIBS is indicated by the red plus signs in Table 1 and gas chromatograms are shown in Supporting Information File 1, Figures S2 and S3.

**Table 1:** Enzymatic synthesis of analogs of **1**^a^.

substrates	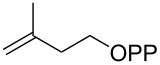 IPP	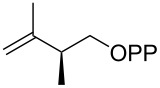 **IA-1**	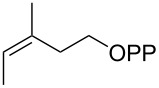 **IA-2**	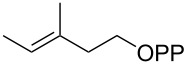 **IA-3**

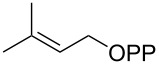 DMAPP	+++	+++	+	++
	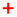	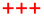		
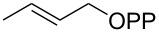 **DA-1**				
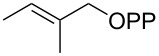 **DA-2**			+	
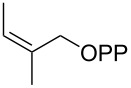 **DA-3**			++	
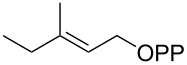 **DA-4**		+	+	+
		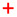		
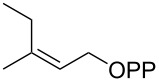 **DA-5**	+	++	+++	+++
		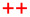		
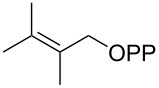 **DA-6**	+		+++	+++

^a^Production of GPP analogs from DMAPP and IPP analogs with FPPS determined by dephosphorylation and GC–MS and IPP analogs with FPPS and 2MIBS is indicated by red plus signs. +++ = high production (peak abundance in the GC MSD ChemStation software >10^7^), ++ = medium production (peak abundance 10^6^ – 10^7^), + = low production (peak abundance <10^6^).

**Scheme 2:**
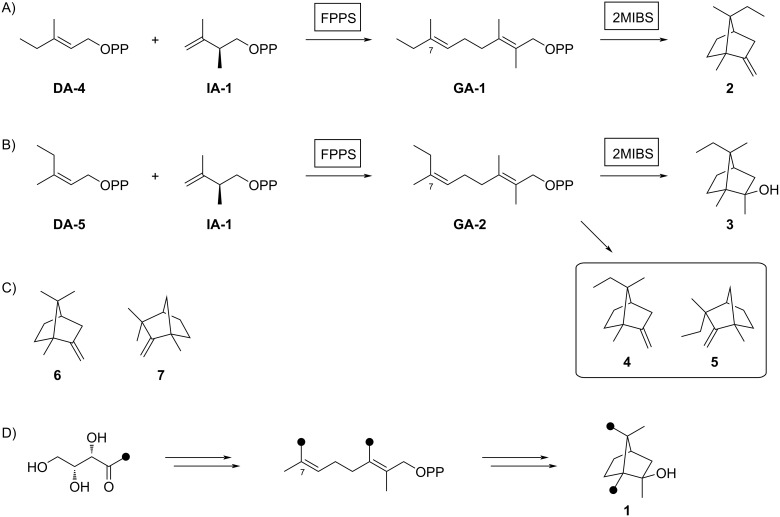
Enzymatic synthesis of analogs of **1**. A) Preparation of **2** from **DA-4** and **IA-1**, B) preparation of **3** and the inseparable mixture of **4** and **5** from **DA-5** and **IA-1**, C) structures of 2MIBS side products **6** and **7**, and D) the stereochemical course for the geminal methyl groups in GPP investigated by feeding of (1-^13^C)-1-desoxyxylulose. Black dots represent ^13^C-labelled carbons.

After identification of the successful substrate-enzyme combinations, a preparative scale incubation of **DA-4** and **IA-1** with FPPS and 2MIBS resulted in the production of a terpenoid hydrocarbon that was isolated and structurally characterised through NMR spectroscopy (Supporting Information File 1, Table S2, Figures S4–S11) as compound **2** (Scheme 2A), a homolog of the 2MIBS side product 2-methylenebornane (**6**) (Scheme 2C). Analogously, the preparative scale conversion of **DA-5** and **IA-1** with FPPS and 2MIBS allowed for the isolation of the 2-methylisoborneol homolog **3** (Scheme 2B, Supporting Information File 1, Table S3 and Figures S12–S19). Furthermore, two inseparable hydrocarbons were obtained as a mixture (8:3) whose structures were tentatively assigned based on the NMR spectra (Supporting Information File 1, Tables S4 and S5, Figures S20–S31) as those of **4**, the corresponding homolog of **6**, and **5**, a homolog of the 2MIBS side product 1-methylcamphene (**7**) (Scheme 2C). The stereochemical course for the cyclisation of the GPP analogs **GA-1** and **GA-2** with respect to the face selectivity at C-7 is reflected by the positioning of the ethyl groups in the products **2** and **3**. Notably, the findings correspond to those made with the native substrate 2-Me-GPP in feeding experiments with (1-^13^C)-1-deoxyxylulose [30].

## Conclusion

In the present study we have investigated two aspects of 2-methylisoborneol biosynthesis. First, the function of the disordered prolin-rich N-terminal domain that is present in many, but not all 2-methylisoborneol synthases was addressed. A comparison between wildtype 2MIBS and a truncated variant with the proline-rich N-terminal domain removed demonstrated a higher activity for the truncated enzyme, while the long-term solubility was better for the full length enzyme, suggesting a stabilising function of the N-terminal domain. Simultaneously, this domain serves as a gatekeeper that extenuates the production of **1**. A second aspect of our study has addressed the substrate scope of FPPS and 2MIBS for the enzymatic synthesis of analogs of **1**. Through this approach two new homologs of 2-methylisoborneol and 2-methylenebornane could be obtained, besides an inseparable mixture of two more tenatively identified compounds. The poor conversion of GPP by 2MIBS demonstrates its strong adaption for the non-canonical substrate 2-Me-GPP, but some flexibility especially for the conversion of substrates slightly larger than 2-Me-GPP can be observed. This may – similar to the recently described biosynthetic machinery for the C_17_ compound chlororaphen that has likely evolved from the C_16_ system for sodorifen [38] – potentially allow for an evolution towards a natural biosynthetic pathway for C_12_ monoterpenoids.

## Experimental

### General synthetic methods

Chemicals were purchased from Sigma-Aldrich Chemie GmbH (Steinheim, Germany), Carbolution Chemicals GmbH (St. Ingbert, Germany), or Carl Roth (Karlsruhe, Germany) and used without purification. Solvents for column chromatography were purchased in p.a. grade and purified by distillation. Thin-layer chromatography was performed with 0.2 mm precoated plastic sheets Polygram Sil G/UV254 purchased from Machery-Nagel (Düren, Germany). Column chromatography was performed using silica gel 60 (0.040–0.060 nm) purchased from Merck (Darmstadt, Germany). NMR spectra were recorded on a Bruker Avance I 500 MHz spectrometer and a Bruker Avance III HD 700 MHz Cryo spectrometer. Chemical shifts were referenced to the residual proton signal of C_6_D_6_ (δ = 7.16 ppm) for ^1^H NMR and the ^13^C signal of C_6_D_6_ (δ = 128.06 ppm) for ^13^C NMR [39]. Coupling constants are given in Hz. IR spectra were recorded on a Bruker α infrared spectrometer with a diamond ATR probehead. Peak intensities are given as s (strong), m (medium), w (weak) and br (broad). Optical rotations were recorded on a Modular Compact Polarimeter MCP 100 (Anton Paar, Graz, Austria). The temperature setting was 20 °C, the wavelength of the light used was 589 nm (sodium D line), the path-length was 10 cm, and compound concentrations *c* are given in g 100 mL^−1^.

### Synthesis of 2-Me-GPP

To an Et_2_O (10 mL, 0 °C) solution of (*E*)-2,3,7-trimethylocta-2,6-dien-1-ol [22] (500 mg, 2.97 mmol, 1.0 equiv) was added PBr_3_ (322 mg, 1.19 mmol, 0.4 equiv) dropwise. The mixture was stirred at 0 °C for 45 min, and then quenched by pouring onto ice-water (20 mL). The aqueous phase was extracted with Et_2_O (3 × 20 mL). The combined extracts were dried with MgSO_4_ and concentrated under reduced pressure to afford the bromide, which was used for phosphorylation without purification.

(NBu_4_)_3_HP_2_O_7_ (4.02 g, 4.46 mmol, 1.5 equiv) was dissolved in acetonitrile (1 mL), and the bromide (mixed with 10 mL acetonitrile) was added dropwise. The mixture was stirred overnight and the solvent was removed under reduced pressure. The resulting residue was dissolved in aqueous NH_4_HCO_3_ solution (0.25 M) and loaded onto a DOWEX 50WX8 ion-exchange column (NH_4_^+^ form, pH 7.0). The column was flushed slowly with 1.5 column volumes of NH_4_HCO_3_ buffer (25 mM, 5% iPrOH) and the eluate was lyophilised to yield 2-Me-GPP (800 mg, 2.11 mmol, 71%) as a white powder.

^1^H NMR (500 MHz, D_2_O) δ 5.30–5.21 (m, 1H), 4.48 (d, *J* = 5.0, 2H), 2.15–2.12 (m, 4H), 1.79 (d, *J* = 1.6, 3H), 1.77 (d, *J* = 1.5, 3H), 1.71 (d, *J* = 1.3, 3H), 1.65 (d, *J* = 1.3, 3H) ppm; ^13^C NMR (126 MHz, D_2_O) δ 135.80 (C_q_), 133.72 (C_q_), 125.03 (d, ^3^*J*_C,P_ = 8.5, CH), 124.26 (CH), 67.10 (d, ^2^*J*_C,P_ = 5.6, CH_2_), 34.11 (CH_2_), 25.67 (CH_2_), 24.83 (CH_3_), 17.41 (CH_3_), 16.86 (CH_3_), 15.67 (CH_3_) ppm; ^31^P NMR (202 MHz, D_2_O) δ −7.90 (d, ^2^*J*_P,P_ = 21.3), −10.40 (d, ^2^*J*_P,P_ = 21.4) ppm; HRMS–TOF (*m*/*z*): calc. for [C_11_H_21_O_7_P_2_]^–^ 327.0768; found, 327.0762.

### Synthesis of (*Z*)-2-methylbut-2-en-1-yl diphosphate (**DA-3**)

The same procedure was used to convert (*Z*)-2-methylbut-2-en-1-ol [35] (292 mg, 3.40 mmol) into **DA-3** (880 mg, 2.96 mmol, 87%) that was obtained as a white powder. ^1^H NMR (500 MHz, D_2_O) δ 5.44 (q, ^3^*J*_H,H_ = 6.8, 1H), 4.40 (d, ^3^*J*_H,P_ = 5.9, 2H), 1.69 (m, 3H), 1.58 (dm, ^3^*J*_H,H_ = 7.0, 3H) ppm; ^13^C NMR (126 MHz, D_2_O) δ 132.34 (d, ^3^*J*_C,P_ = 8.0 Hz, C_q_), 124.64 (CH), 64.18 (d, ^2^*J*_C,P_ = 5.3, CH_2_), 20.52 (d, ^4^*J*_C,P_ = 1.8, CH_3_), 12.62 (d, ^5^*J*_C,P_ = 2.2, CH_3_) ppm; ^31^P NMR (202 MHz, D_2_O) δ −7.0 (d, ^2^*J*_P,P_ = 21.2 Hz), −10.2 (d, ^2^*J*_P,P_ = 21.4 Hz) ppm.

### Synthesis of (*E*)-3-methylpent-2-en-1-yl diphosphate (**DA-4**)

The same procedure was used to convert (*E*)-3-methylpent-2-en-1-ol [36] (800 mg, 8.00 mmol) into **DA-4** (2.30 g, 7.40 mmol, 92%) that was obtained as a white powder. ^1^H NMR (500 MHz, D_2_O) δ 5.36 (t, ^3^*J*_H,H_ = 7.3 Hz, 1H), 4.38 (t, ^3^*J*_H,H_ = 6.8 Hz, 2H), 1.98 (q, ^3^*J*_H,H_ = 7.5 Hz, 2H), 1.63 (s, 3H), 0.92 (t, ^3^*J*_H,H_ = 7.5 Hz, 3H) ppm; ^13^C NMR (126 MHz, D_2_O) δ 145.09 (C_q_), 118.13 (d, ^3^*J*_C,P_ = 8.5 Hz, CH), 62.46 (d, ^2^*J*_C,P_ = 5.2 Hz, CH_2_), 31.60 (CH_2_),15.58 (CH_3_), 11.62 (CH_3_) ppm; ^31^P NMR (202 MHz, D_2_O) δ −6.6 (d, ^2^*J*_P,P_ = 21.4 Hz), −10.2 (d, ^2^*J*_P,P_ = 21.4 Hz) ppm.

### Synthesis of (*Z*)-3-methylpent-2-en-1-yl diphosphate (**DA-5**)

The same procedure was used to convert (*Z*)-3-methylpent-2-en-1-ol [37] (215 mg, 2.15 mmol) into **DA-5** (604 mg, 1.94 mmol, 90%) that was obtained as a white powder. ^1^H NMR (500 MHz, D_2_O) δ 5.38 (t, ^3^*J*_H,H_ = 6.5 Hz, 1H), 4.41 (t, ^3^*J*_H,H_ = 6.8 Hz, 2H), 2.09 (q, ^3^*J*_H,H_ = 7.6 Hz, 2H), 1.72 (s, 3H), 0.94 (t, ^3^*J*_H,H_ = 7.6 Hz, 3H) ppm; ^13^C NMR (126 MHz, D_2_O) δ 145.52 (C_q_), 119.35 (d, ^3^*J*_C,P_ = 8.4 Hz, CH), 62.05 (d, ^2^*J*_C,P_ = 5.2 Hz, CH_2_), 24.53 (CH_2_), 22.12 (CH_2_), 12.44 (CH_3_) ppm; ^31^P NMR (202 MHz, D_2_O) δ −6.4 (d, ^2^*J*_P,P_ = 21.9 Hz), −10.3 (d, ^2^*J*_P,P_ = 21.9 Hz) ppm.

## Supporting Information

File 1 Biosynthesis and enzymatic preparation of the non-natural analogs, analytical data and spectra.
