# Supplementary material for: Functions of enzyme domains in 2-methylisoborneol biosynthesis and enzymatic synthesis of non-natural analogs
Source: Beilstein J Org Chem. 2023 Sep 22;19:1452–9. doi: 10.3762/bjoc.19.104 (PMC10520479; doi:10.3762/bjoc.19.104)
Supplement: File 1 — Biosynthesis and enzymatic preparation of the non-natural analogs, analytical data and spectra. [file Beilstein_J_Org_Chem-19-1452-s001.pdf]

BEILSTEIN JOURNAL OF ORGANIC CHEMISTRY

Supporting Information

for

Functions of enzyme domains in 2-methylisoborneol biosynthesis and enzymatic synthesis of non-natural analogs

Binbin Gu, Lin-Fu Liang and Jeroen S. Dickschat

Beilstein J. Org. Chem. **2023,** *19,* 1452–1459. doi:10.3762/bjoc.19.104

Biosynthesis and enzymatic preparation of the non-natural analogs, analytical data and spectra

License and Terms: This is a supporting information file under the terms of the Creative Commons Attribution License (https://creativecommons.org/licenses/by/4.0). Please note that the reuse, redistribution and reproduction in particular requires that the author(s) and source are credited and that individual graphics may be subject to special legal provisions.
The license is subject to the *Beilstein Journal of Organic Chemistry* terms and conditions: (https://www.beilstein-journals.org/bjoc/terms)

Gene cloning

Following a published procedure [1], the target genes of domain A and domain B from *Streptomyces coelicolor* A3(2) were amplified from gDNA by PCR using Q5 High-fidelity DNA polymerase (New England Biolabs, Ipswich, MA, USA) and primer pairs as listed in Table S1. Yeast homologous recombination of the PCR products with the linearised pYE-Express shuttle vector [2] were carried out through the standard protocol using LiOAc, polyethylene glycol and salmon sperm DNA [3]. After yeast transformation cultures were grown on SM-URA agar (425 mg yeast nitrogen base, 1.25 g ammonium sulfate, 5 g glucose, 192.5 mg nutritional supplement minus uracil, 5 g agar, 250 mL water) at 28 °C for 3 days. The recombinant plasmids were isolated from grown yeast colonies using the Zymoprep Yeast Plasmid Miniprep II kit (Zymo Research, Irvine, CA, USA) and subsequently used for transformation of *Escherichia coli* BL21(DE3) electrocompetent cells. Cells were plated on LB agar plates with kanamycin (50 µg mL^{-1}) followed by incubation at 37 °C overnight. Single colonies were selected and used to inoculate LB medium (6 mL) liquid cultures with kanamycin (6 µL; 50 mg mL^{-1}). After 24 h growth the plasmid DNA was isolated and checked for correct insertion of the desired gene by PCR amplifying the DNA sequence, containing the target gene, using the T7 primer pair and by sequencing. The obtained plasmids were named pYE-Domain A and pYE-Domain B. Construction of the pYE-Express plasmid containing the gene for 2MIBS from *S. coelicolor* A3(2) (pYE-WP_011031839) was reported before [4].

Table S1. Primers used for cloning of the coding genes for 2MIBS, domain A and domain B.

**Primer**[a]	**Sequence**[b]
Domain A_F	GGCAGCCATATGGCTAGCATGACTGGTGGAatgcccgactccgggaccctcggaac
Domain A_R	TCTCAGTGGTGGTGGTGGTGGTGCTCGAGTGctaggcggcgggcggagcggatg
Domain B_F	GGCAGCCATATGGCTAGCATGACTGGTGGAggacgagccgtccccggc
Domain B_R	TCTCAGTGGTGGTGGTGGTGGTGCTCGAGTGctaccagaagtcgggcaggctgtagc

[a] Primer names are composed of the accession numbers and “F” for forward or “R” for reverse complement primer. [b] The sequence shown in capital letters are homology arms for recombination in yeast which match the terminal sequences of the linearised expression vector pYE-Express (HindIII and EcoRI digestion).

Gene expression and protein purification

Following a published procedure [1], precultures of *E. coli* BL21 (DE3) transformed with pYE-Domain A (Domain A), pYE-Domain B (Domain B) and pYE-WP_011031839 (2MIBS) were grown in LB medium with kanamycin (50 µg mL^{-1}) overnight with shaking at 37 °C. Gene expression cultures were inoculated with the precultures (2/100) and grown in LB medium containing kanamycin (50 µg mL^{-1}) with shaking at 37 °C until OD_{600} = 0.4–0.6 was reached. After cooling the cultures to 18 °C the enzyme expression was induced by the addition of aqueous IPTG solution (400 mM, 1/1000). The cultures were shaken at 18 °C overnight. The cells were harvested via centrifugation (10000g, 5 min, 4 °C), resuspended in binding buffer (10 mL L^{-1} culture; 20 mM Na_2HPO_4, 500 mM NaCl, 20 mM imidazole, 1 mM $MgCl_2$, pH 7.4, 4 °C) and lysed by ultrasonication (8 × 1 min) on ice. The cell debris was removed by centrifugation (14600g, 10 min, 4 °C) and the soluble protein fractions were loaded onto Ni^{2+}-NTA superflow affinity chromatography columns (Qiagen, Venlo, Netherlands) equilibrated with binding buffer. The columns were washed with washing buffer (2 × 10 mL L^{-1} culture; 20 mM Na_2HPO_4, 500 mM NaCl, 50 mM imidazole, 1 mM $MgCl_2$, pH 7.4, 4 °C) and the desired proteins were eluted with elution buffer (2 × 6.25 mL L^{-1} culture; 20 mM Na_2HPO_4, 500 mM NaCl, 500 mM imidazole, 1 mM $MgCl_2$, pH 7.4, 4 °C). The protein concentrations were determined by Bradford assay [5]. Protein yields were: 2MIBS (2.5 mg mL^{-1}), domain A (1.0 mg mL^{-1}) and domain B (4.0 mg mL^{-1}).

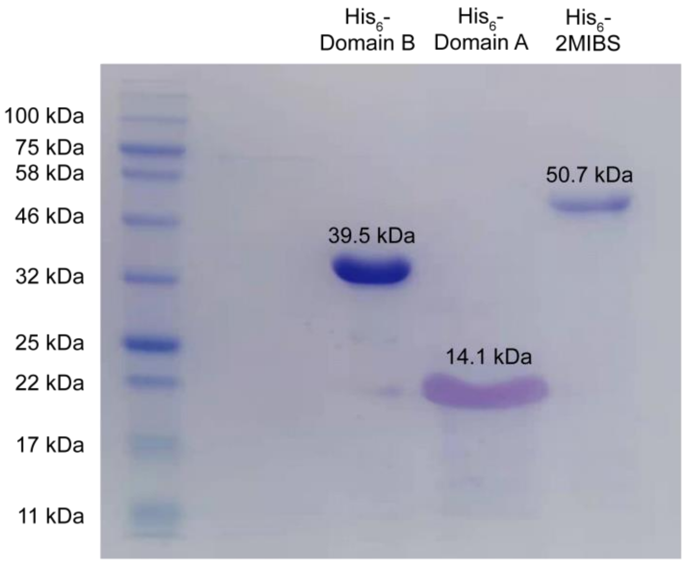

Figure S1. SDS-PAGE analysis of recombinant 2MIBS, domain A and domain B (all enzymes carry N-terminal His-tags).

Incubation of 2-Me-GPP with 2-MIBS, domain A, domain B and domains A + B

Culture conditions, protein expressions and protein purifications were performed as described above. The soluble enzyme fractions were checked for purity by SDS-PAGE. The incubations were performed in incubation buffer (50 mM Tris/HCl, 10 mM $MgCl_2$, 10% glycerol, pH 8.2) with a final reaction volume of 1 mL. 2-Me-GPP (0.3 mg, 0.8 mM) dissolved in 50 µL substrate buffer (25 mM NH_4HCO_3) and the enzyme(s) (2MIBS, domain A, domain B, or domain A + domain B; 0.3 µM each) were added and the final reaction volumes were adjusted to 1 mL with incubation buffer, followed by incubation with shaking at 30 °C for 12 h. The crude product was extracted with hexane (500 µL), the extract was dried with $MgSO_4$ and directly analysed by GC–MS.

GC–MS and GC/MS-QTOF analyses

GC–MS analyses were carried out on a 7890B/5977A series gas chromatography/mass selective detector (Agilent, Santa Clara, CA, USA). The GC was equipped with an HP5-MS fused silica capillary column (30 m, 0.25 mm i. d., 0.50 µm film; Agilent) and operated using the settings: 1) inlet pressure: 77.1 kPa, He at 23.3 mL min^{-1}, 2) injection volume: 1 µL, 3) temperature program: 5 min at 50 °C then increasing 10 °C min^{-1} to 320 °C, 4) splitless or split ratio 50:1, 60 s valve time, and 5) carrier gas: He at 1 mL min^{-1}. The MS was operated with the same settings: 1) source:

230 °C, 2) transfer line: 250 °C, 3) quadrupole: 150 °C and 4) electron energy: 70 eV. Retention indices (*I*) were determined from a homologous series of *n*-alkanes (C_7–C_{40}). GC–MS-QToF analyses were performed on a 7890B GC equipped with a HP5-MS fused silica capillary column (30 m, 0.25 mm i. d., 0.50 µm film) connected to a 7200 accurate-mass QTOF detector (Agilent). GC parameters were 1) inlet pressure: 83.2 kPa, He at 24.6 mL min^{-1}, 2) injection volume: 1 µL, 3) split ratio: 50:1, 60 s valve time, 4) temperature program: 5 min at 50 °C increasing at 5 °C min^{-1} to 320 °C, 5) carrier gas: He at 1 mL min^{-1}. MS parameters were 1) transfer line: 250 °C, 2) electron energy 70 eV.

Enzyme incubation of different combinations of DMAPP derivatives and IPP derivatives with FPPS and 2MIBS

Following a published procedure [6], Culture conditions, protein expressions and protein purifications were performed as described above. The soluble enzyme fractions were checked for purity by SDS-PAGE. The test incubations were performed with DMAPP derivatives (0.3 mg) and IPP derivatives (0.3 mg) dissolved in substrate buffer (50 µL; 25 mM NH_4HCO_3) and diluted with incubation buffer (700 µL; 50 mM Tris/HCl, 10 mM $MgCl_2$, 10% glycerol, pH 8.2). FPPS and 2MIBS protein solutions (100 µL) obtained from 100 mL expression culture were added to the mixture, followed by incubation with shaking at 30 °C for 12 h. The crude product was extracted with hexane (500 µL), the extract was dried with $MgSO_4$ and directly analysed by GC–MS. Large scale preparations were done by dissolving 30 mg **DA-4** or **DA-5** and 30 mg **IA-1** in substrate buffer (25 mL). These solutions were added to a mixture of FPPS (25 mL; from 8 L expression culture, 2.1 mg mL^{-1}) and 2MIBS (25 mL; from 8 L expression culture, 2.3 mg mL^{-1}) protein preparations and incubation buffer (150 mL). The reaction mixture was stirred overnight at 30 °C. The reaction was extracted with *n*-pentane (3 × 200 mL), and the extracts were dried with $MgSO_4$ and concentrated in vacuo (600 mbar, 35 °C). Column chromatography on silica gel yielded **2** (elution with *n*-pentane, from **DA-4** and **IA-1**), or an inseparable mixture of **4** and **5** (elution with *n*-pentane) and **3** (elution with *n*-pentane/Et_2O = 2:1; from **DA-5** and **IA-1**).

Compound 2. TLC [100% *n*-pentane]: R_f = 0.84. Yield: 0.4 mg, 2.4 µmol, 3%, from **DA-4** (30 mg, 96 µmol) and **IA-1** (30 mg, 96 µmol). GC (HP-5MS): *I* = 1138. MS (EI, 70 eV): *m*/*z* (%) = 164 (11), 149 (16), 135 (32), 121 (16), 107 (100), 95 (31), 94 (39), 93 (74), 91 (76), 82 (12), 79 (74), 77 (29), 67 (24), 55 (30), 53 (19), 41 (53), 39 (23).

IR (diamond ATR): ṽ = 2950 (m), 2924 (s), 2854 (m), 1728 (w), 1658 (w), 1632 (w), 1510 (w), 1459 (m), 1410 (w), 1379 (w), 1259 (m), 1185 (w), 1092 (s), 1013 (s), 873 (w), 796 (s), 702 (w) cm^{-1}. HR-MS (Q-TOF, 70 eV): calc. $[C_{12}H_{20}]^+$ *m*/*z* = 164.1560; found: *m*/*z* = 164.1558. Optical rotary power: $[\alpha]_D^{20}$ = –7.5 (*c* 0.04, CH_2Cl_2). NMR data are given in Table S2.

Compound 3. TLC [*n*-pentane/Et_2O (2:1)]: R_f = 0.55. Yield: 1.0 mg, 5.5 µmol, 6%, from **DA-5** (30 mg, 96 µmol) and **IA-1** (30 mg, 96 µmol). GC (HP-5MS): *I* = 1306. MS (EI, 70 eV): *m*/*z* (%) = 182 (1), 164 (5), 153 (17), 135 (19), 125 (9), 124 (9), 109 (29), 107 (27), 95 (100), 85 (24), 83 (17), 81 (22), 79 (12), 69 (11), 67 (20), 57 (12), 55 (22), 43 (39), 41 (16), 39 (9). IR (diamond ATR): ṽ = 3324 (s), 2958 (s), 2925 (s), 2873 (s), 1682 (s), 1607 (m), 1510 (w), 1459 (w), 1386 (s), 1300 (s), 1260 (w), 1189 (w), 1179 (w), 1097 (m), 1047 (w), 1014 (w), 799 (s), 665 (w), 588 (s), 570 (s), 544 (s) cm^{-1}. HR-MS (Q-TOF, 70 eV): calc. $[C_{12}H_{22}O]^+$ *m*/*z* = 182.1665; found: *m*/*z* = 182.1664. Optical rotary power: $[\alpha]_D^{20}$ = +4.0 (*c* 0.1, CH_2Cl_2). NMR data are given in Table S3.

Inseparable mixture of 4 and 5. TLC [100% *n*-pentane]: R_f = 0.75. Yield: 1.0 mg, 6.1 µmol, 6%, from **DA-5** (30 mg, 96 µmol) and **IA-1** (30 mg, 96 µmol). GC (HP-5MS): *I* = 1139 (**4**), 1104 (**5**). MS (EI, 70 eV, **4**): *m*/*z* (%) = 164 (10), 149 (18), 135 (41), 121 (16), 107 (100), 95 (45), 94 (42), 93 (76), 91 (66), 82 (12), 79 (78), 77 (29), 67 (23), 55 (30), 53 (17), 41 (39), 39 (22). MS (EI, 70 eV, **5**): *m*/*z* (%) = 164 (33), 149 (10), 135 (59), 121 (22), 107 (100), 93 (77), 91 (77), 79 (67), 77 (38), 67 (30), 65 (17), 55 (39), 53 (23), 41 (47), 39 (27). HR-MS (Q-TOF, 70 eV): calc. $[C_{12}H_{20}]^+$ *m*/*z* = 164.1560 for **4** and **5**; found: *m*/*z* = 164.1557 for **4**, *m*/*z* = 164.1558 for **5**. NMR data are given in Tables S4 and S5.

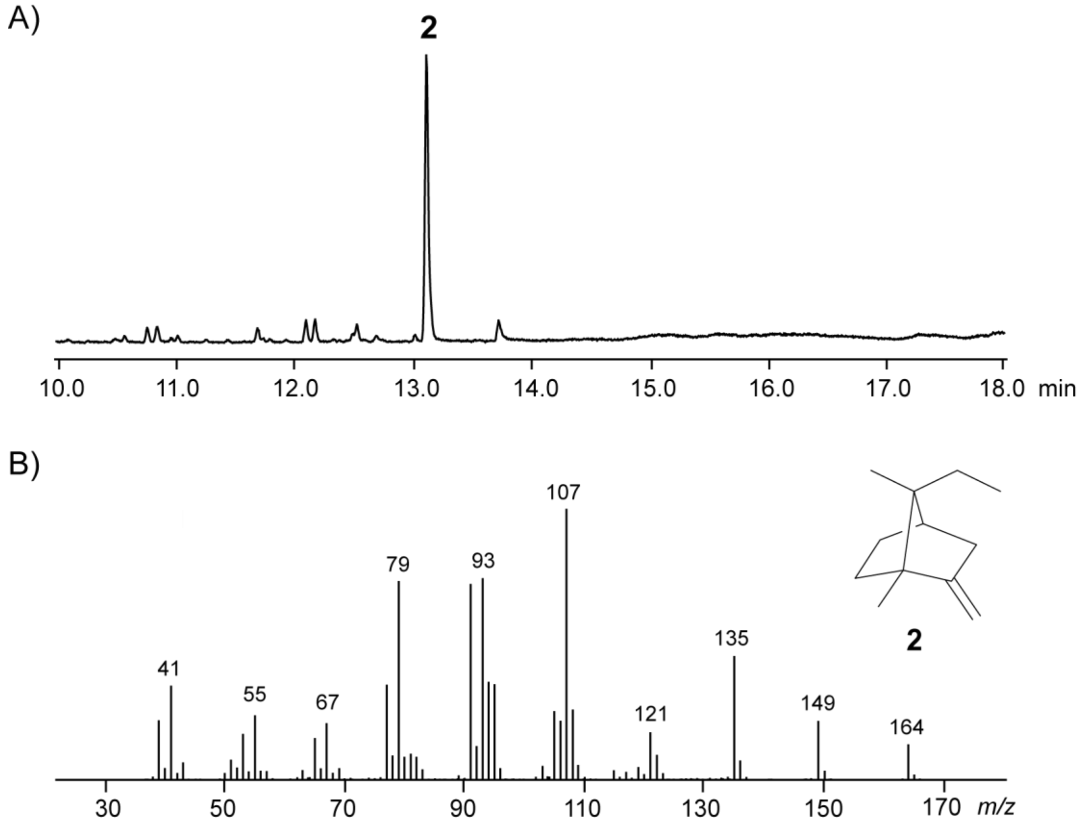

Figure S2: A) Total ion chromatogram (TIC) of products obtained by incubation of **DA-4** and **IA-1** with FPPS and 2MIBS. B) EI-MS spectrum of product **2**.

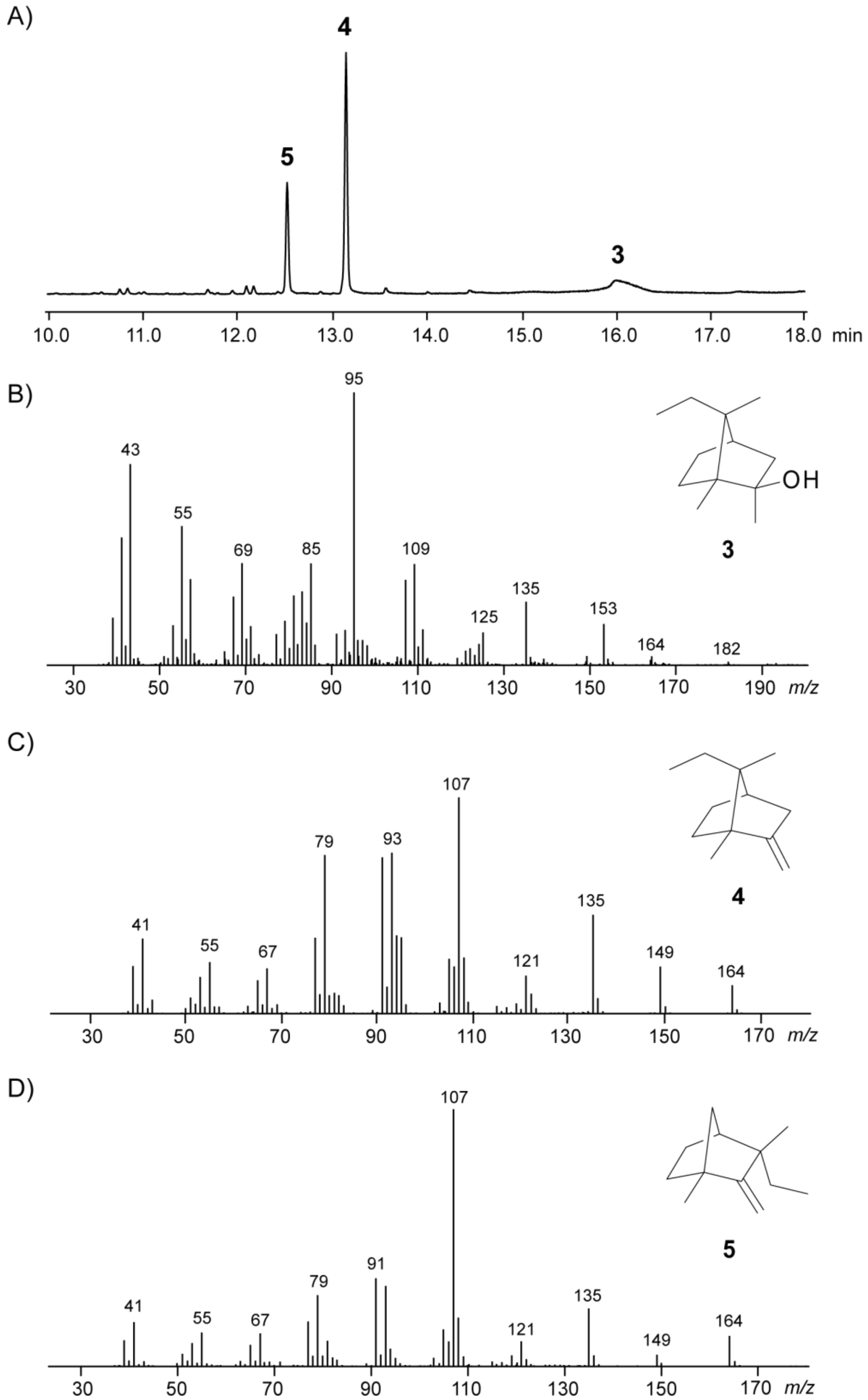

Figure S3. A) Total ion chromatogram (TIC) of products obtained by incubation of **DA-5** and **IA-1** with FPPS and 2MIBS. EI-MS spectra of B) product **3**, C) product **4**, and D) product **5**.

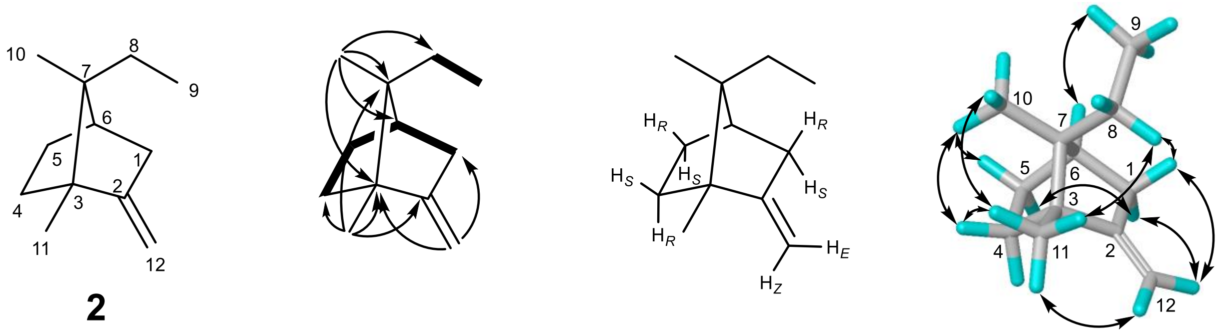

2

Figure S4. Structure elucidation of compound **2**. Bold: ^{1}H,^{1}H-COSY, single headed arrows: key HMBC, and double headed arrows: NOESY correlations. Diastereotopic hydrogens are labelled H_R (*pro-R*), H_S (*pro-S*), H_E (*E*) and H_Z (*Z*).

Table S2. NMR data of **2** in C_6D_6 recorded at 298 K.

		**2**	
C[a]	type	^{13}C[b]	^{1}H[b]
1	CH_2	36.85	2.24 (ddq, *J* = 16.2, 4.9, 2.5, H_R)
			1.85 (dt, *J* = 16.2, 2.2, H_S)
2	C_q	159.68	–
3	C_q	52.60	–
4	CH_2	35.83	1.61 (m, H_S)
			1.34 (m, H_R)
5	CH_2	28.15	1.61 (m, H_R)
			1.13 (m, H_S)
6	CH	41.10	1.74 (t, *J* = 4.0)
7	C_q	50.87	–
8	CH_2	25.84	1.28 (m)
			0.93 (m)
9	CH_3	9.86	0.80 (t, *J* = 7.5)
10	CH_3	15.19	0.76 (d, *J* = 0.8)
11	CH_3	12.68	0.92 (s)
12	CH_2	101.70	4.86 (t, *J* = 2.6, H_Z)
			4.79 (m, H_E)

[a] Carbon numbering as shown in Figure S4. [b] Chemical shifts δ in ppm; multiplicity: s = singlet, d = doublet, t = triplet, q = quartet, m = multiplet; coupling constants *J* are given in Hertz.

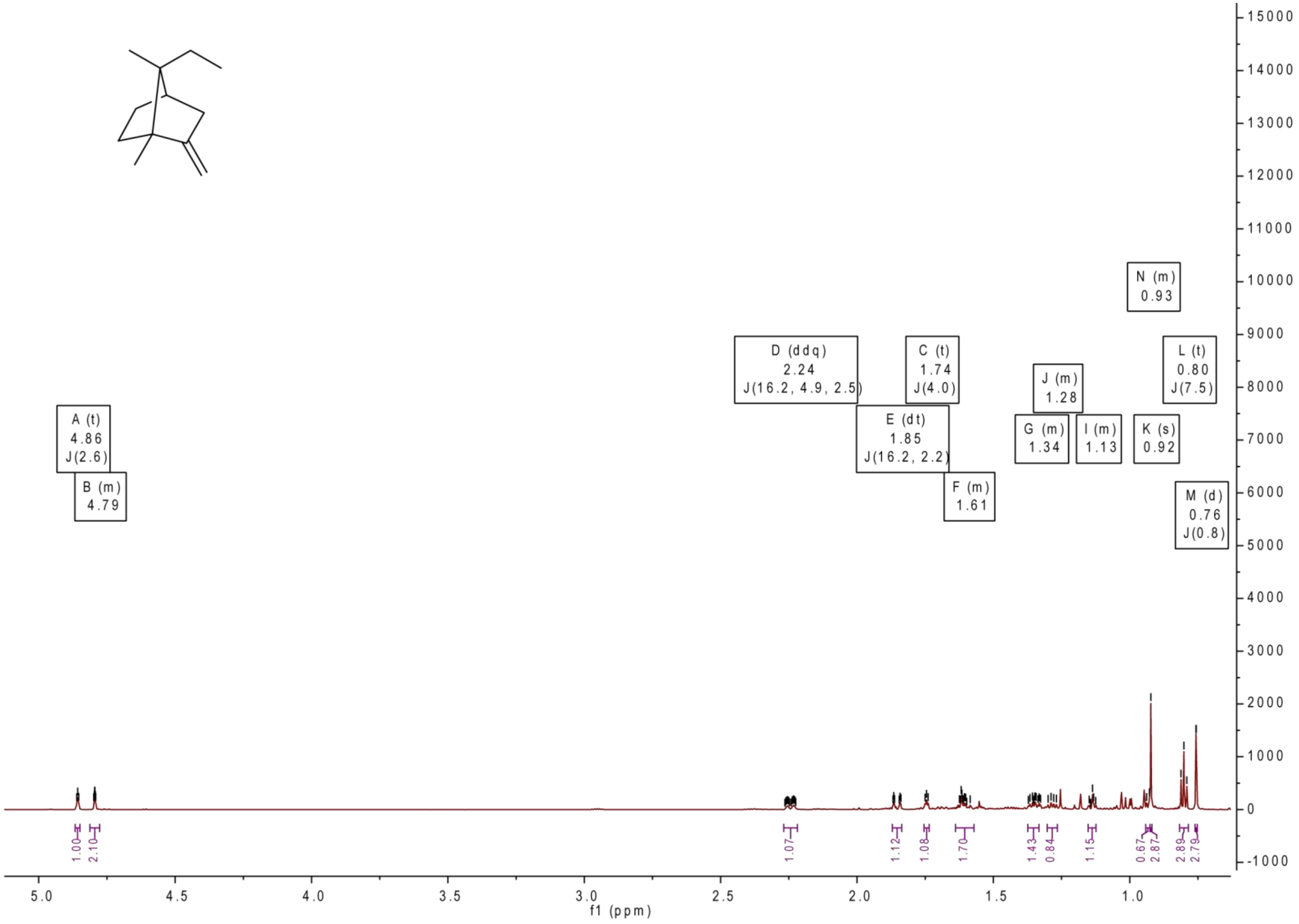

Figure S5. ^{1}H NMR spectrum of **2** (700 MHz, C_6D_6).

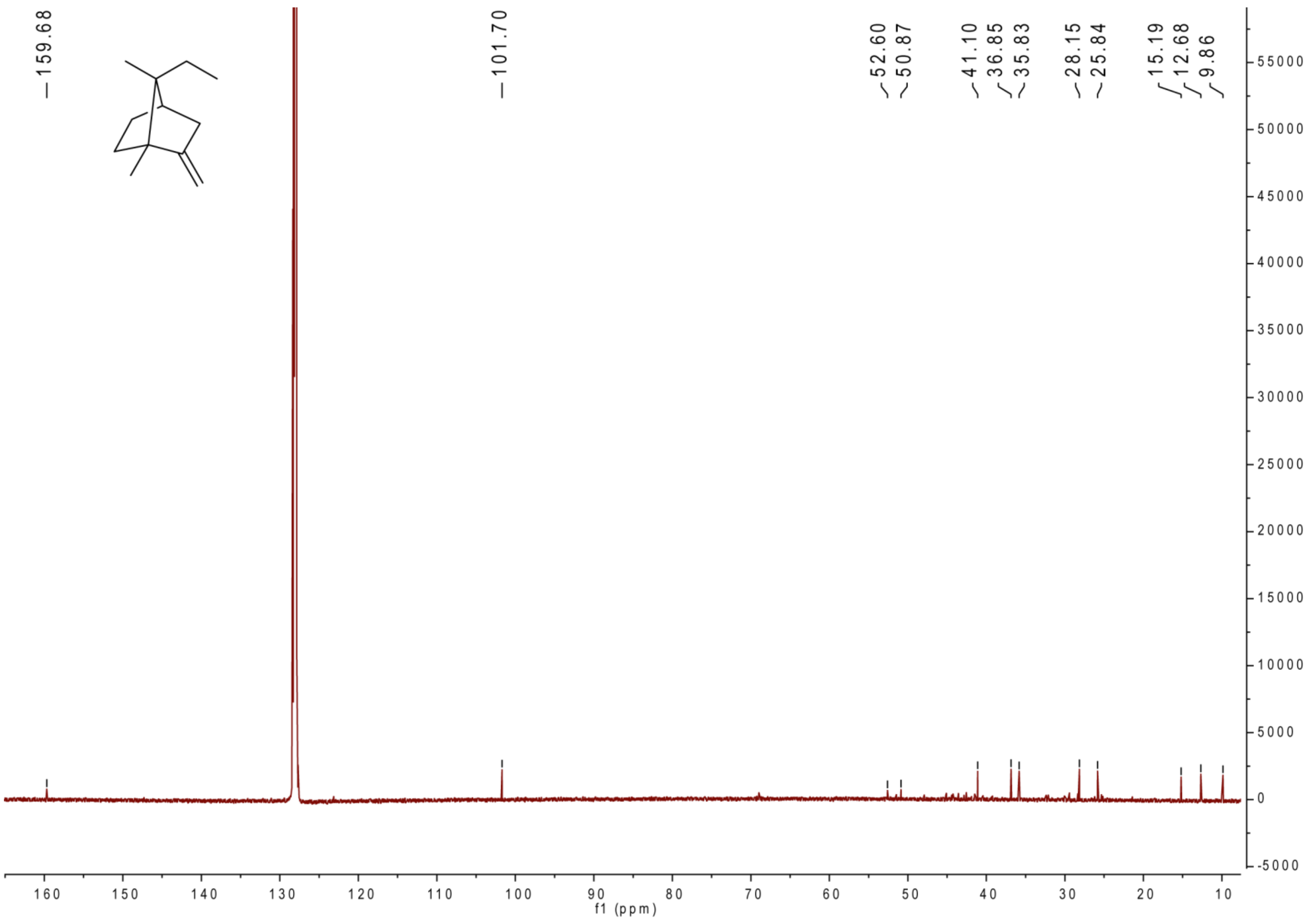

Figure S6. ^{13}C NMR spectrum of **2** (176 MHz, C_6D_6).

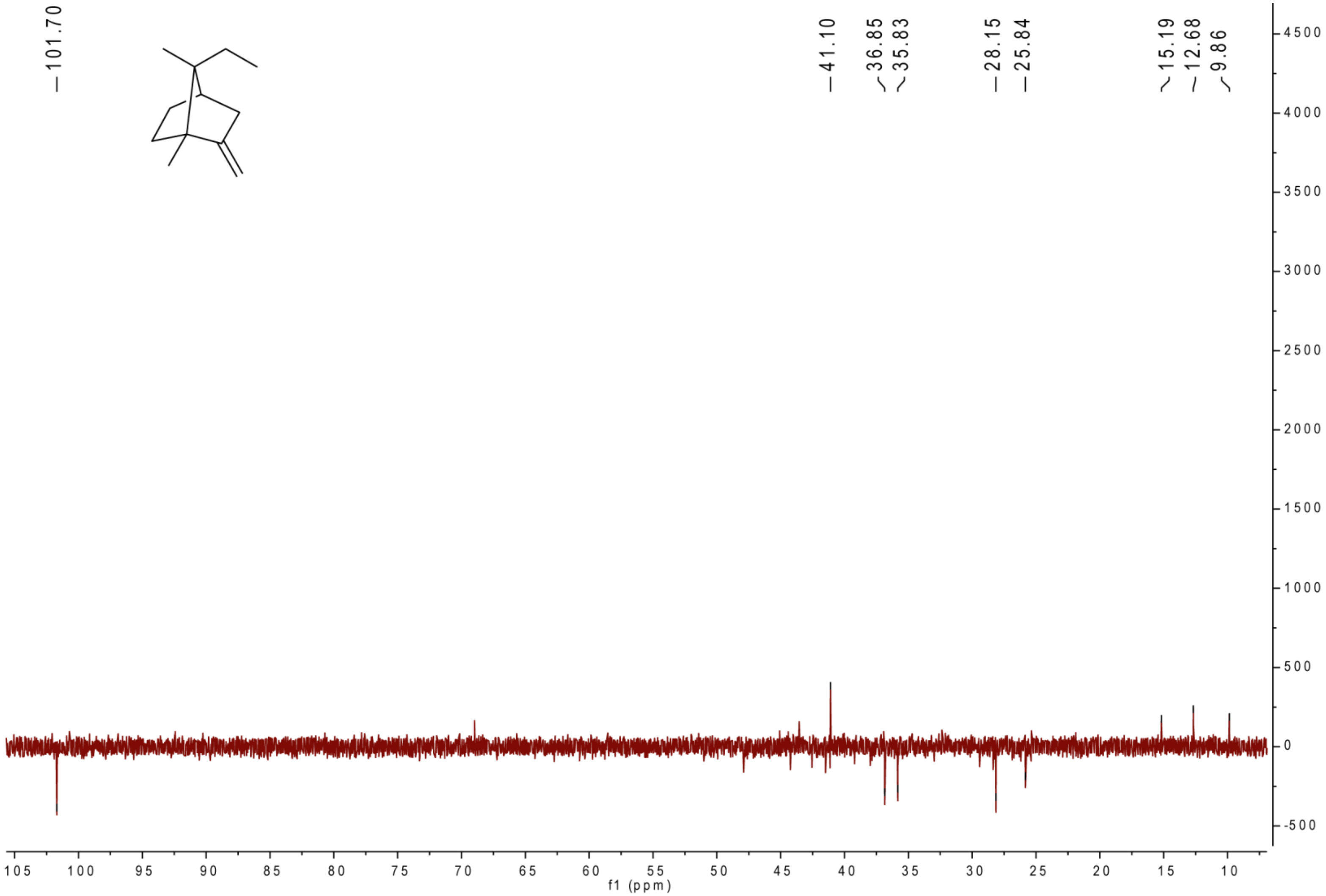

Figure S7. ^{13}C-DEPT135 spectrum of **2** (176 MHz, C_6D_6).

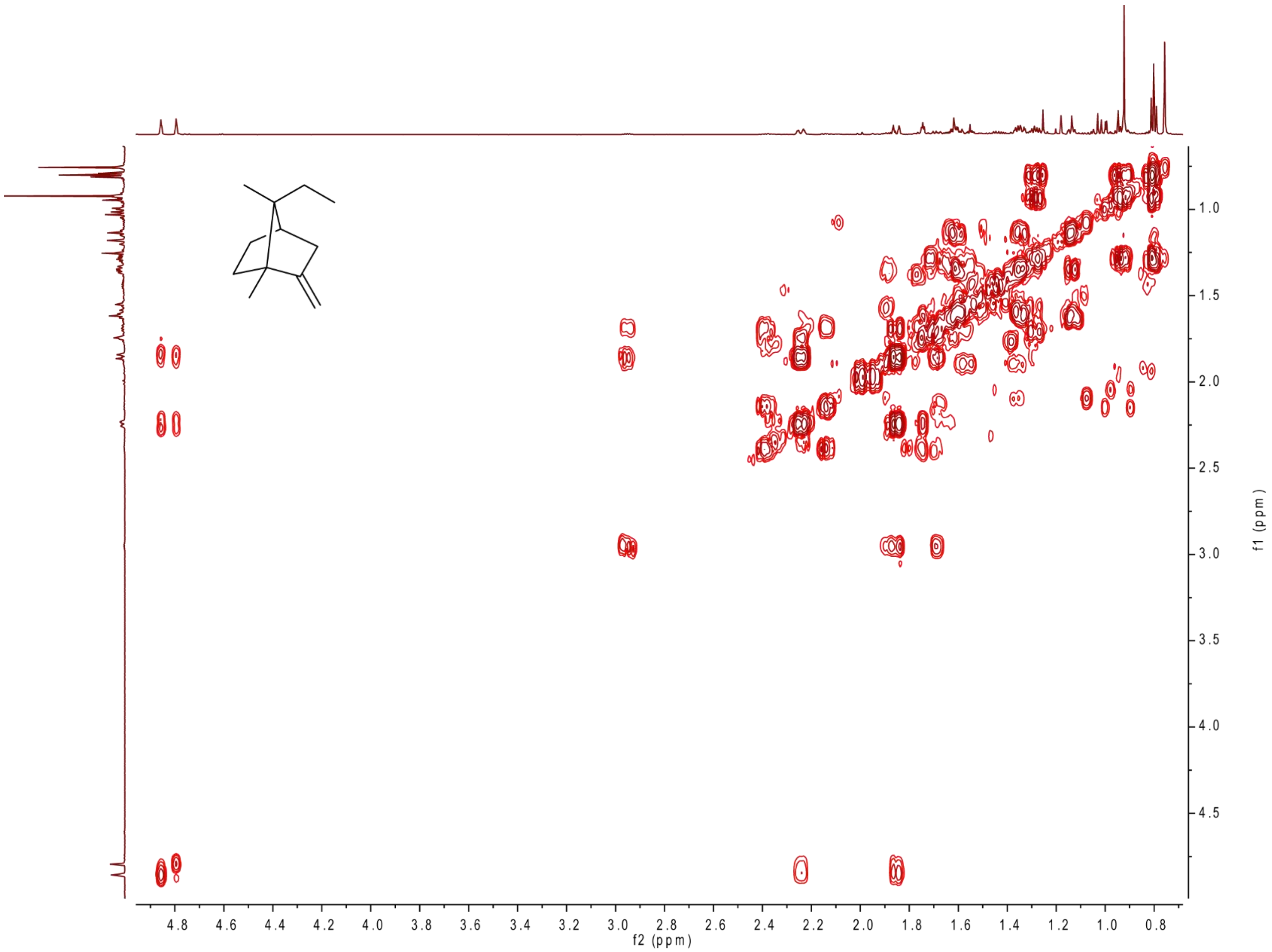

Figure S8. ^{1}H,^{1}H-COSY spectrum (C_6D_6) of **2**.

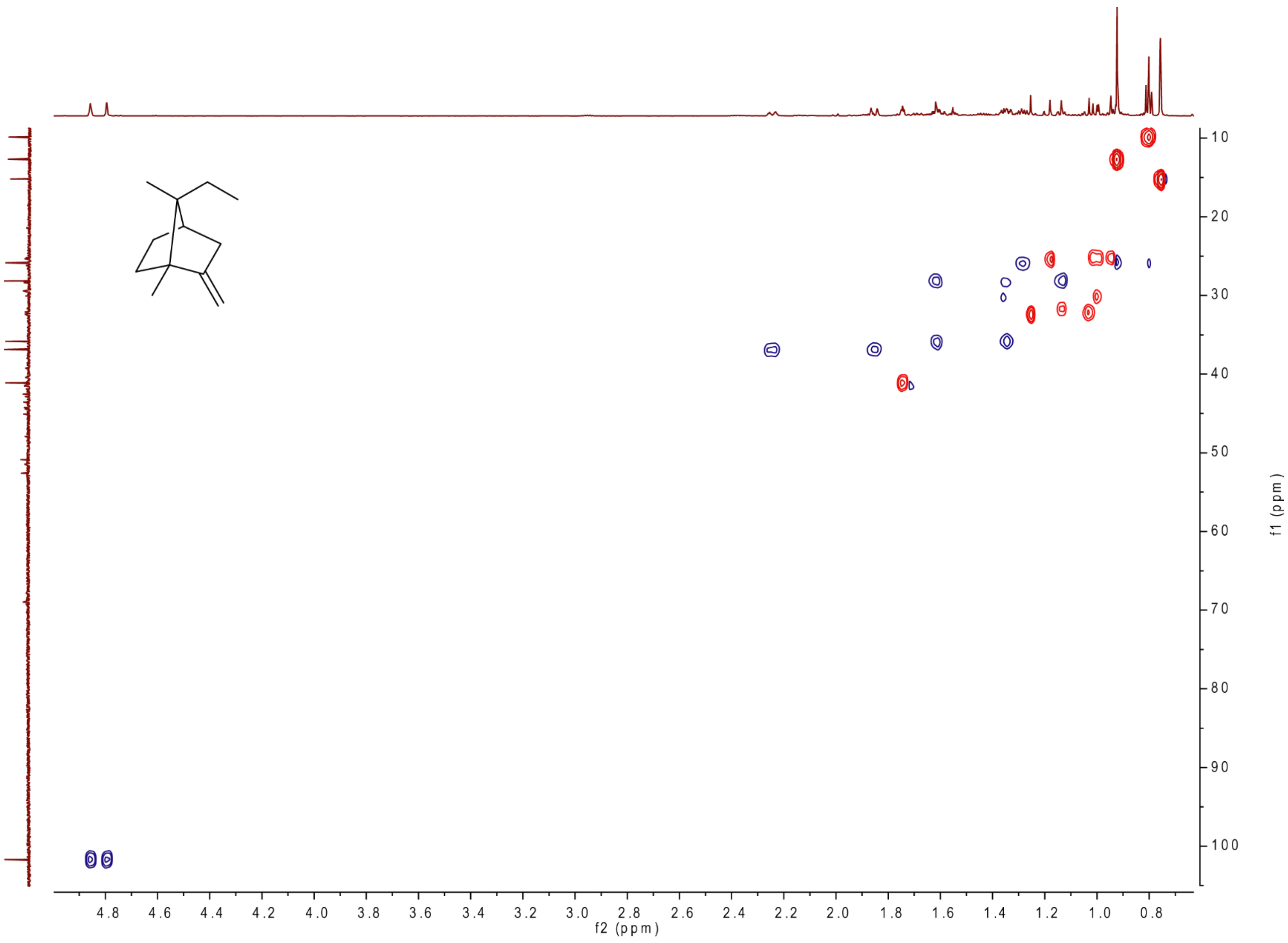

Figure S9. HSQC spectrum (C_6D_6) of **2**.

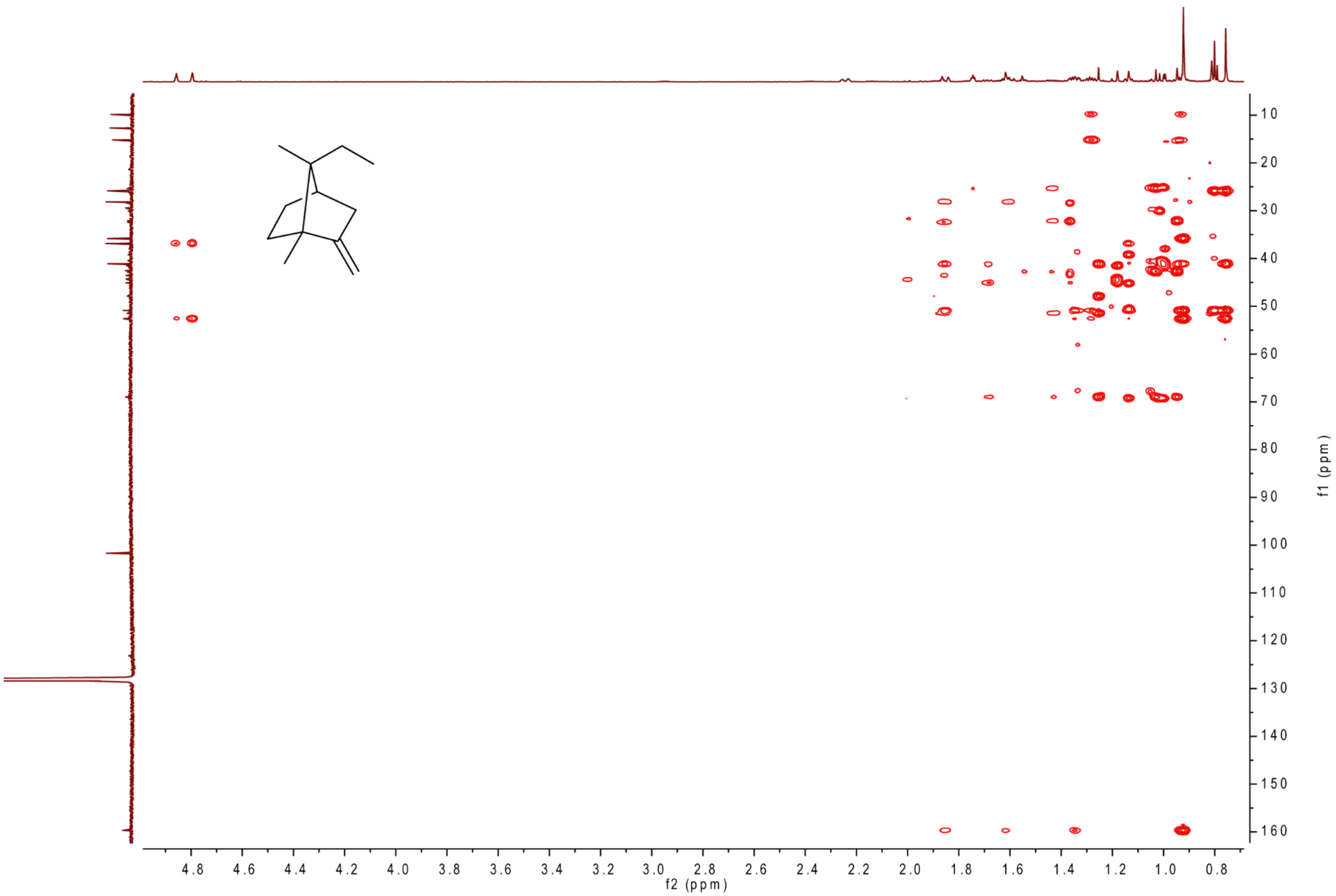

Figure S10. HMBC spectrum (C_6D_6) of **2**.

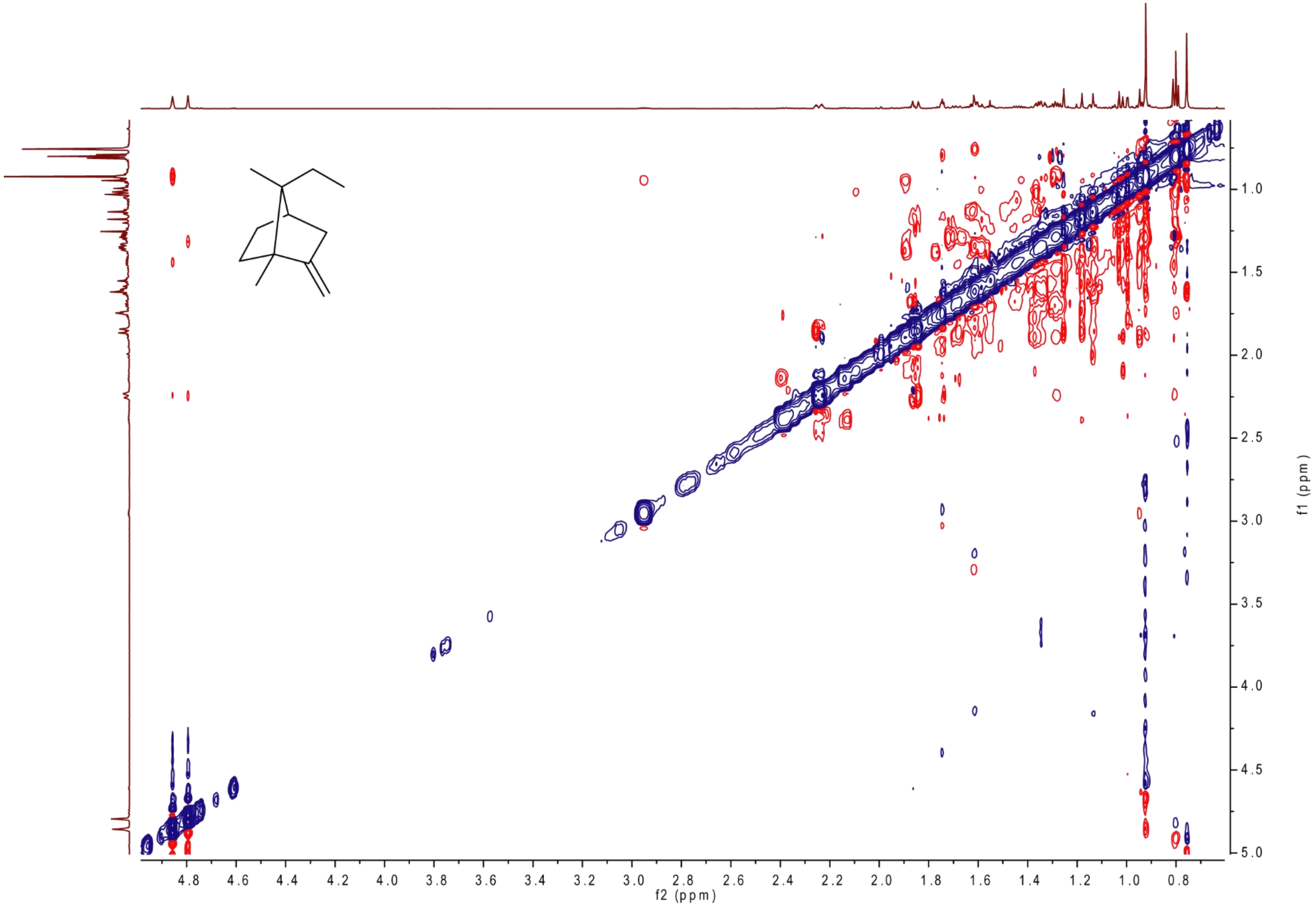

Figure S11. NOESY spectrum (C_6D_6) of **2**.

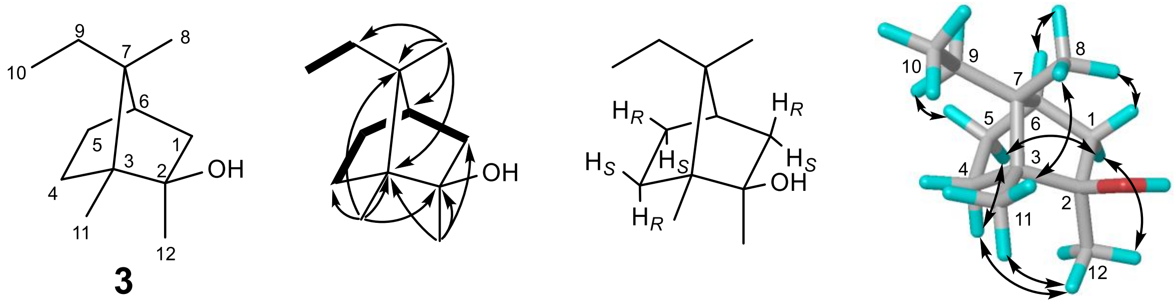

3

Figure S12. Structure elucidation of compound **3**. Bold: ^{1}H,^{1}H-COSY, single headed arrows: key HMBC, and double headed arrows: NOESY correlations. Diastereotopic hydrogens are labelled H_R (*pro-R*) and H_S (*pro-S*).

Table S3. NMR data of **3** in C_6D_6 recorded at 298 K.

		**3**	
C[a]	type	^{13}C[b]	^{1}H[b]
1	CH_2	47.52	2.04 (ddd, *J* = 13.1, 4.5, 3.2, H_R)
			1.22 (d, *J* = 13.1, H_S)
2	C_q	79.49	–
3	C_q	52.85	–
4	CH_2	31.60	1.34 (m, H_S)
			1.26 (ddd, *J* = 13.3, 9.3, 3.7, H_R)
5	CH_2	26.91	1.48 (m, H_R)
			0.87 (m, H_S)
6	CH	42.48	1.73 (t, *J* = 4.5)
7	C_q	52.58	–
8	CH_3	17.32	1.23 (d, *J* = 1.0)
9	CH_2	26.21	1.34 (m)
			0.99 (m)
10	CH_3	9.61	0.86 (t, *J* = 7.4)
11	CH_3	10.28	0.79 (s)
12	CH_3	27.28	1.00 (s)

[a] Carbon numbering as shown in Figure S12. [b] Chemical shifts δ in ppm; multiplicity: s = singlet, d = doublet, t = triplet, m = multiplet; coupling constants *J* are given in Hertz.

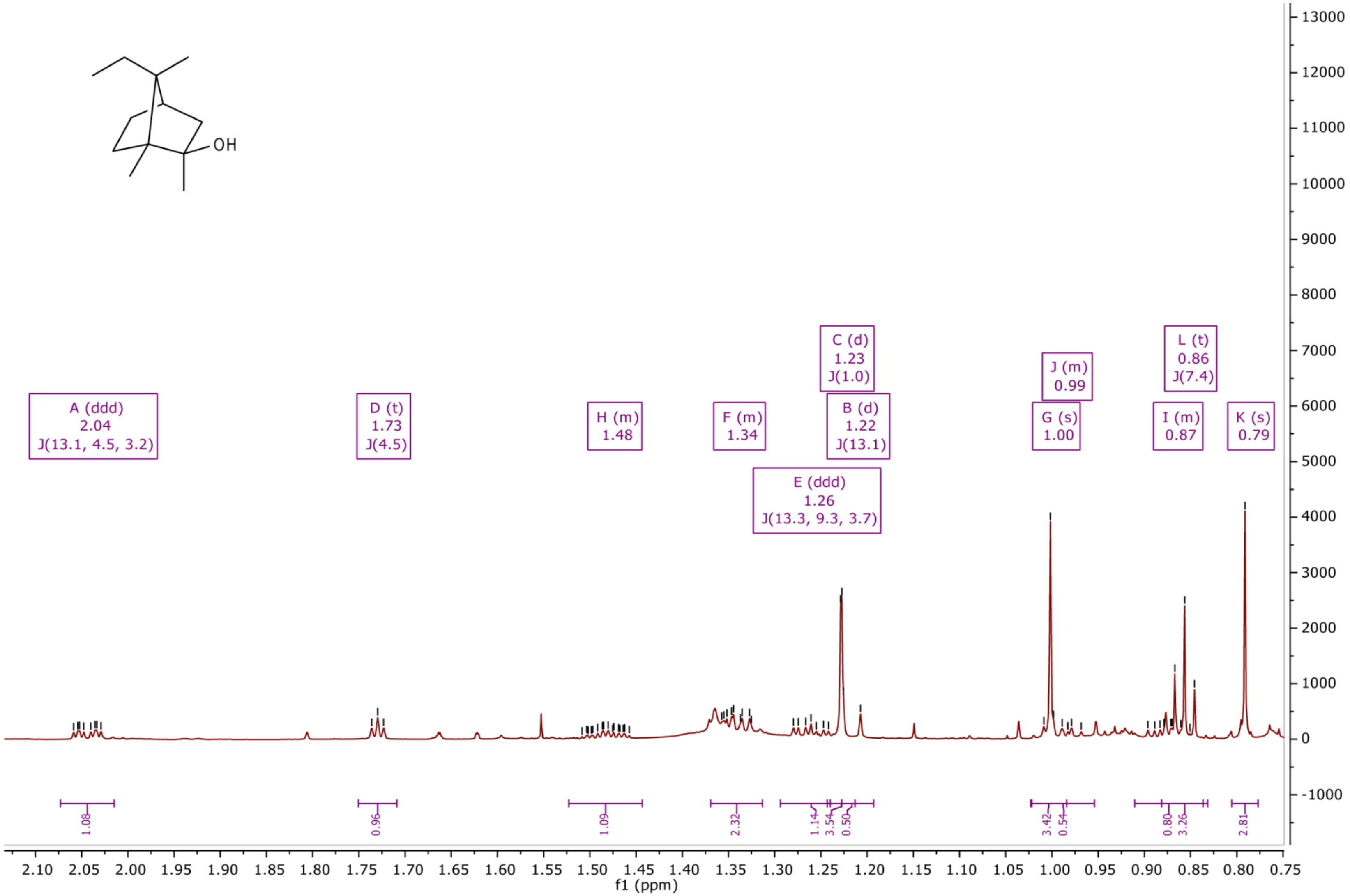

Figure S13. ^{1}H NMR spectrum of **3** (700 MHz, C_6D_6).

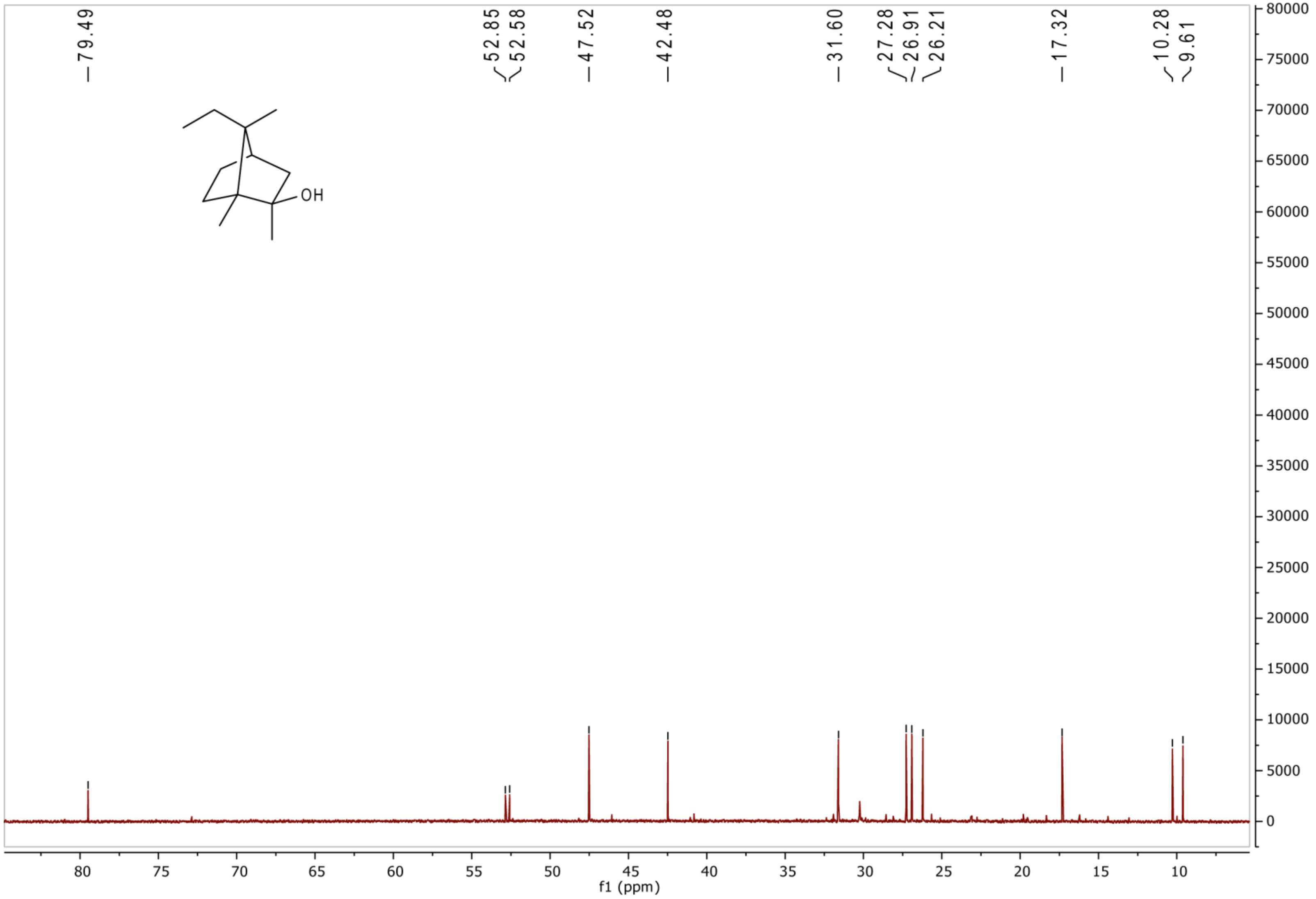

Figure S14. ^{13}C NMR spectrum of **3** (176 MHz, C_6D_6).

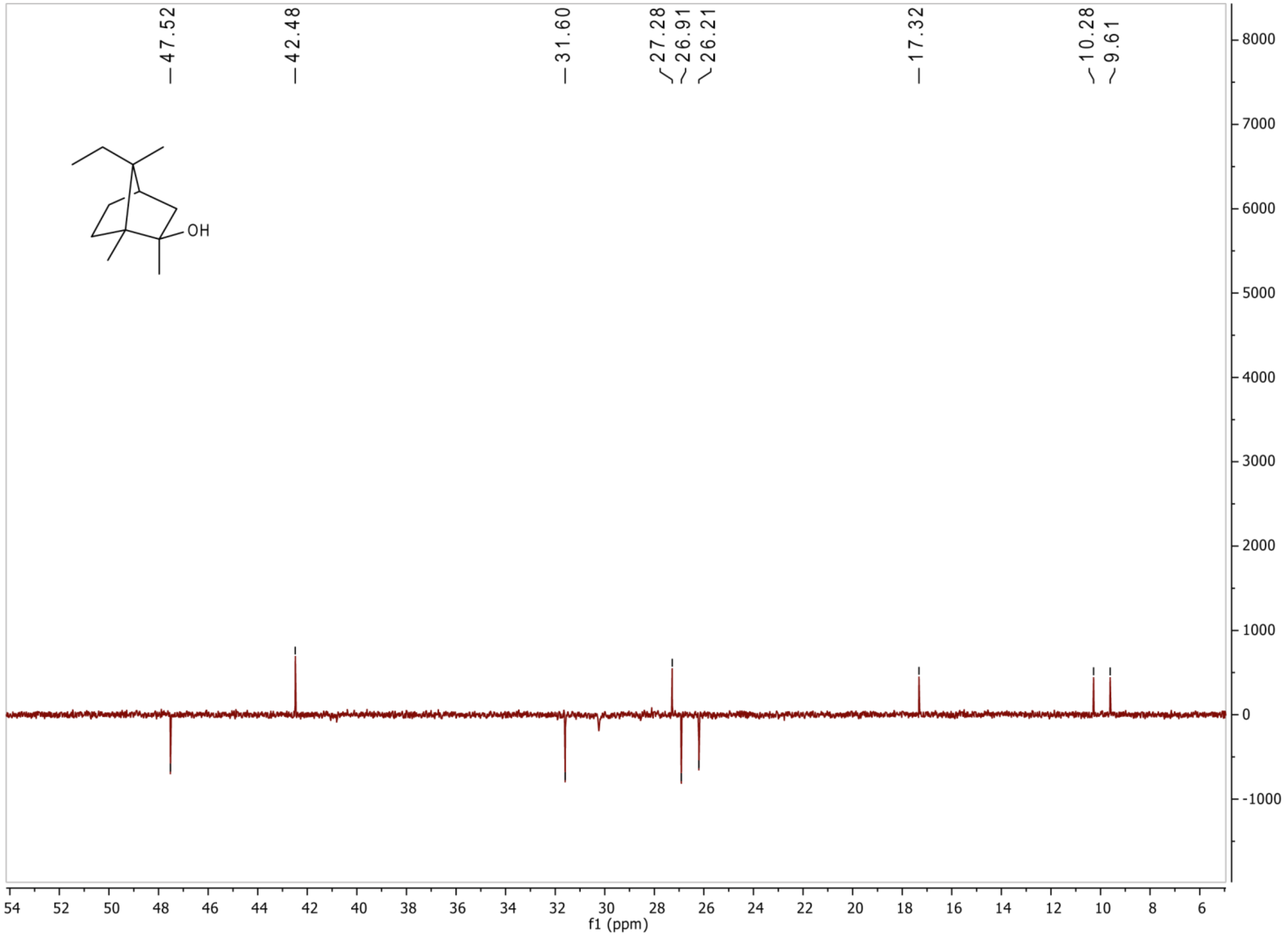

Figure S15. ^{13}C-DEPT135 spectrum of **3** (176 MHz, C_6D_6).

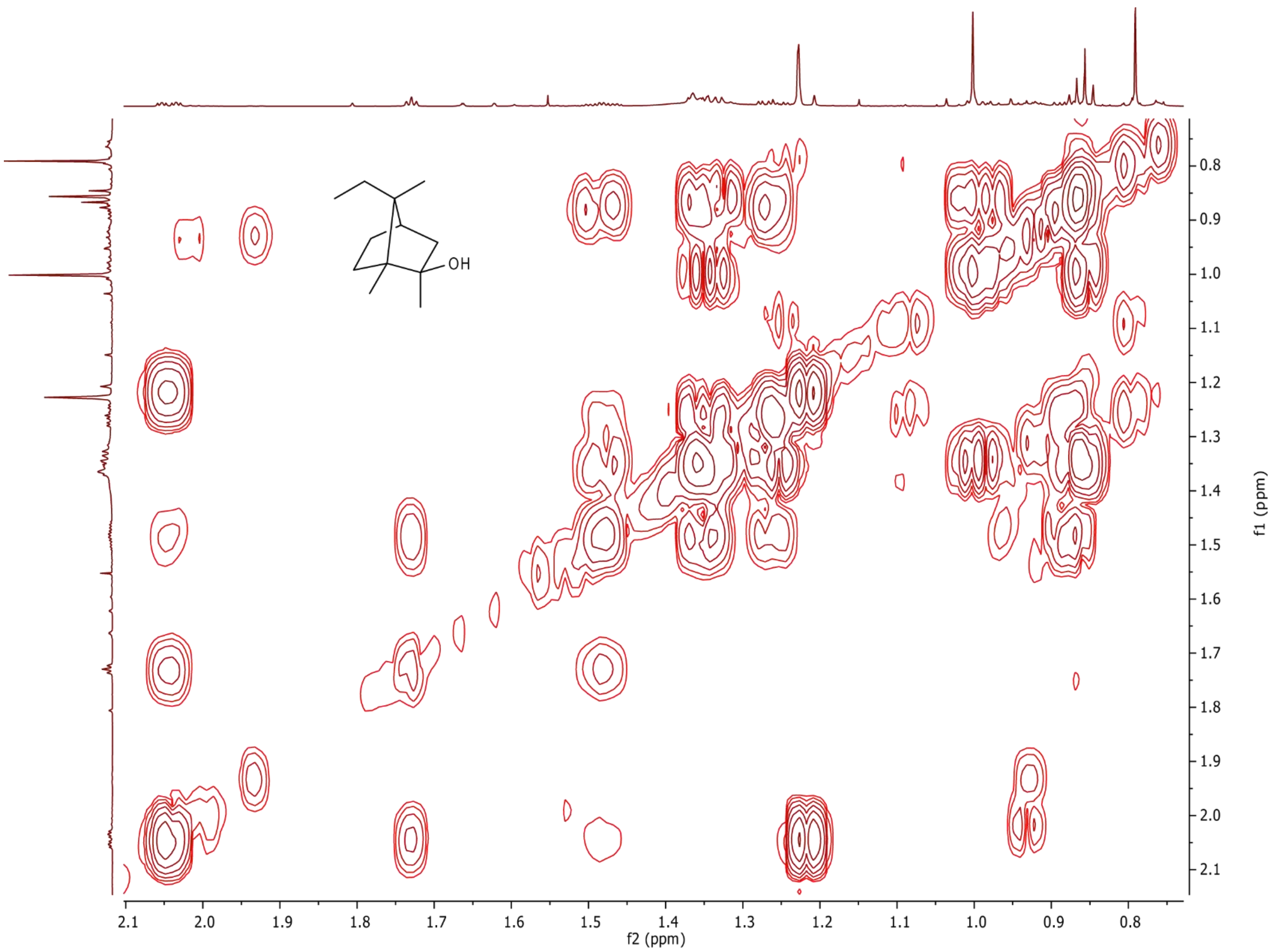

Figure S16. $^{1}H,^{1}H$-COSY spectrum (C_6D_6) of **3**.

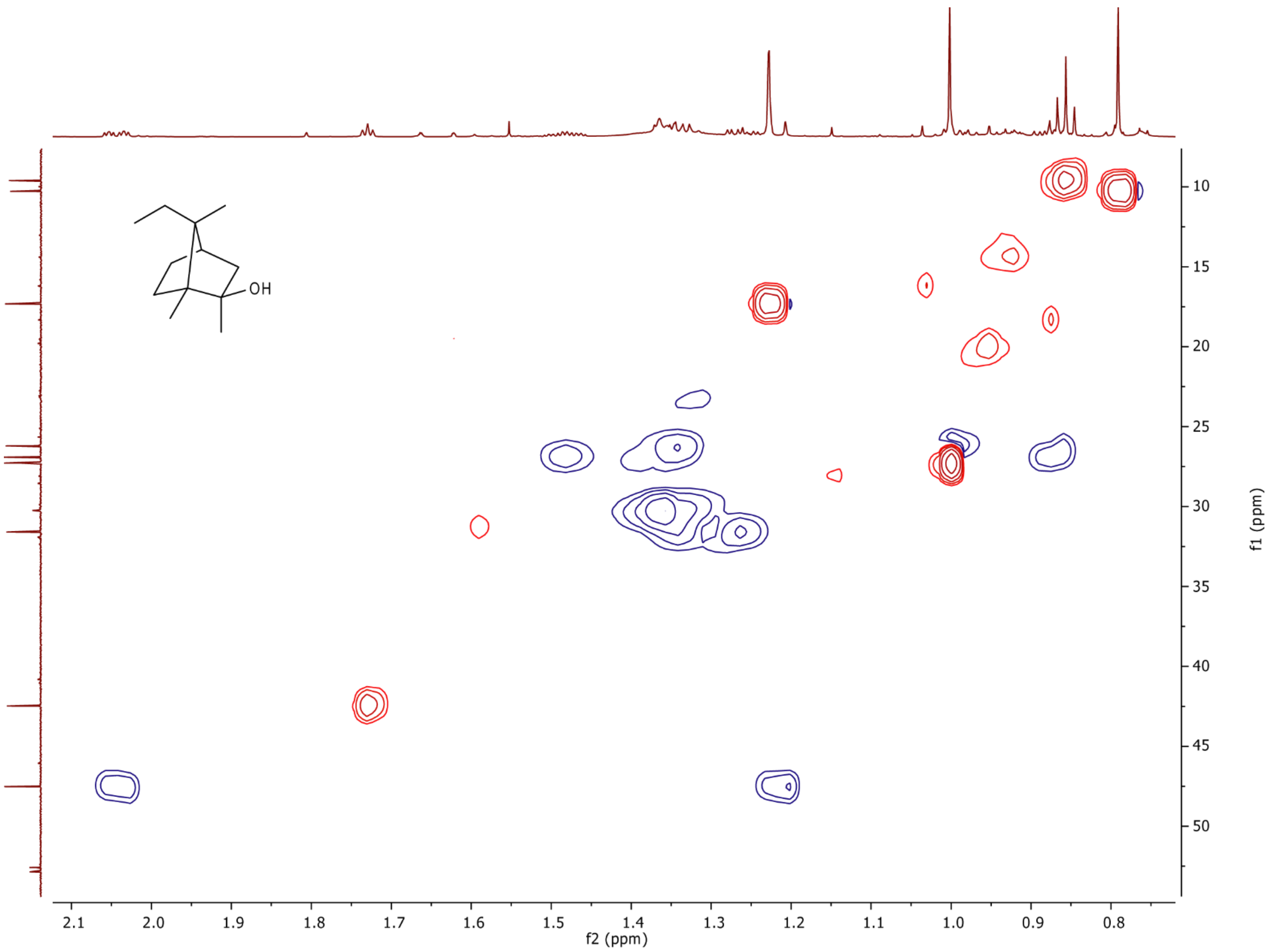

Figure S17. HSQC spectrum (C_6D_6) of **3**.

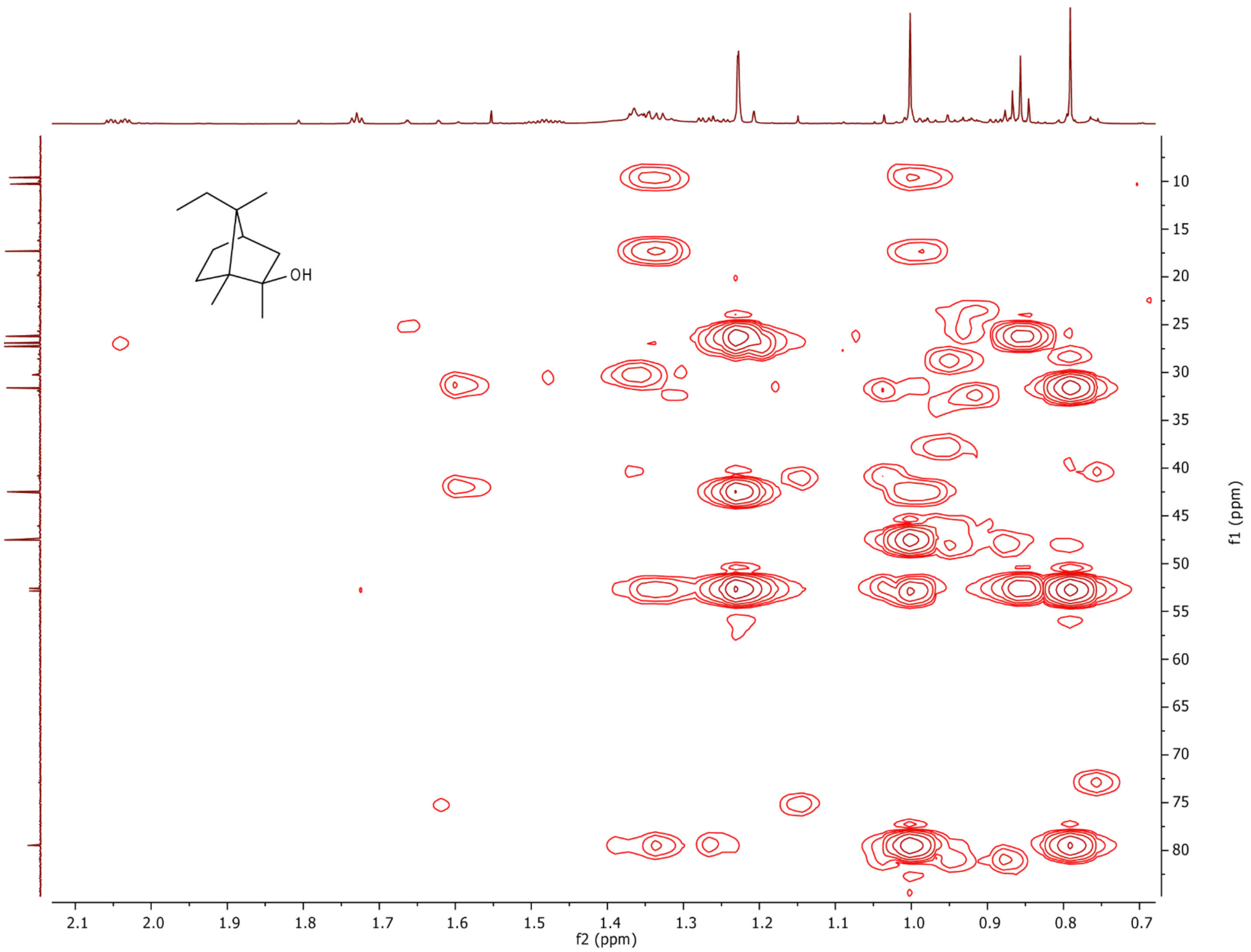

Figure S18. HMBC spectrum (C_6D_6) of **3**.

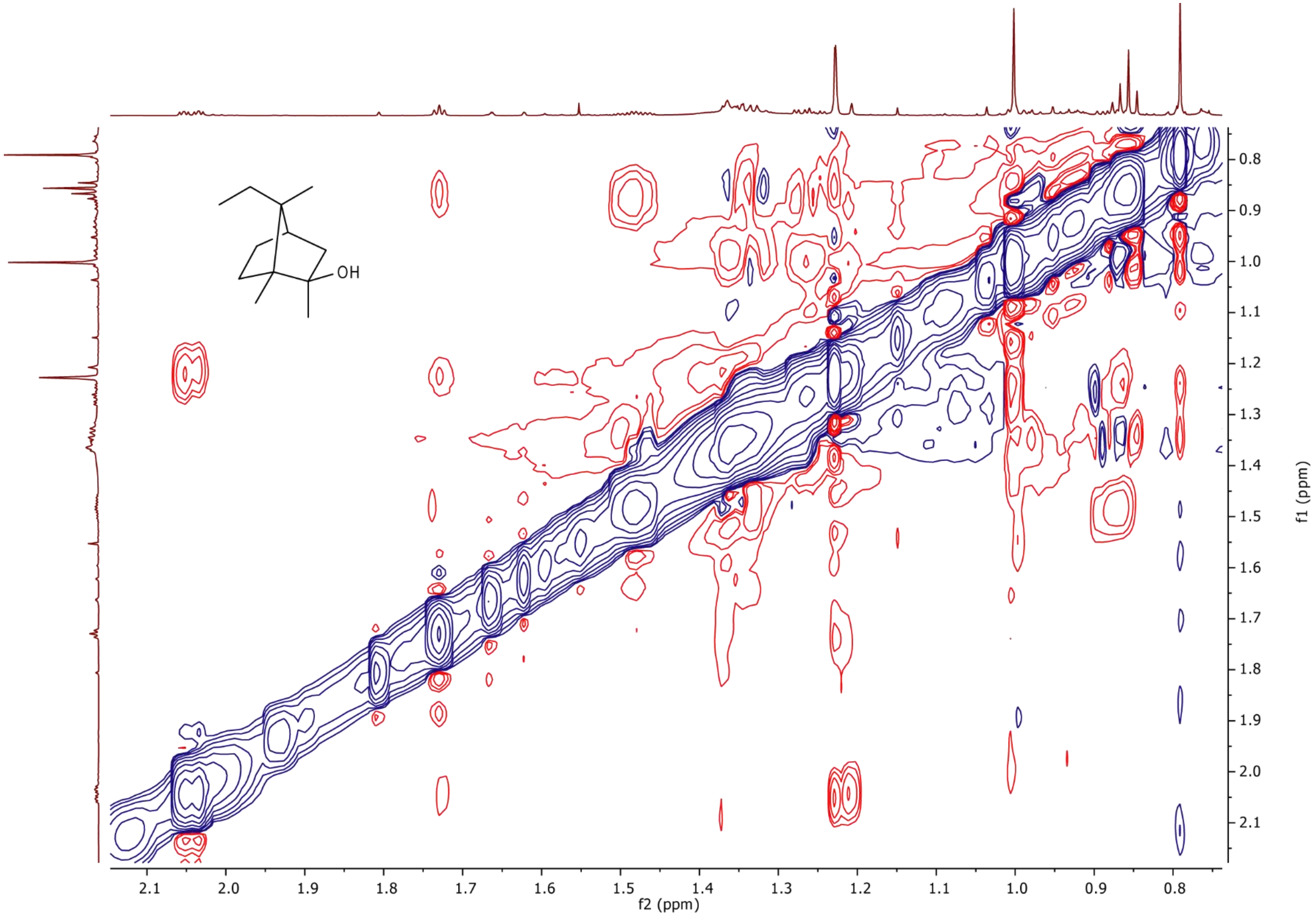

Figure S19. NOESY spectrum (C_6D_6) of **3**.

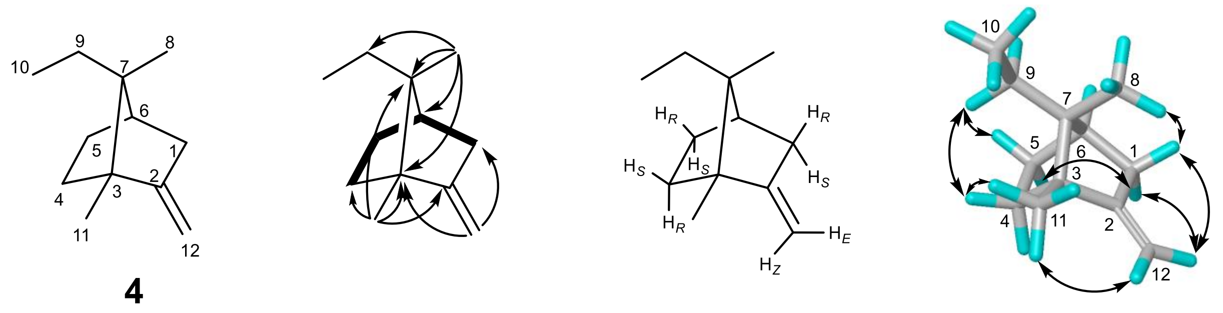

Figure S20. Structure elucidation of compound **4**. Bold: ^{1}H,^{1}H-COSY, single headed arrows: key HMBC, and double headed arrows: NOESY correlations. Diastereotopic hydrogens are labelled H_R (*pro-R*), H_S (*pro-S*), H_E (*E*) and H_Z (*Z*).

Table S4. NMR data of **4** in C_6D_6 recorded at 298 K.

		**4**	
C[a]	type	^{13}C[b]	^{1}H[b]
1	CH_2	36.98	2.29 (ddq, *J* = 16.1, 5.3, 2.6, H_R)
			1.88 (dt, *J* = 16.2, 2.2, H_S)
2	C_q	159.78	–
3	C_q	52.50	–
4	CH_2	35.43	1.61 (m, H_S)
			1.35 (m, H_R)
5	CH_2	27.99	1.58 (m, H_R)
			1.11 (m, H_S)
6	CH	41.33	1.75 (t, *J* = 4.3)
7	C_q	50.76	–
8	CH_3	16.08	0.75 (d, *J* = 0.8)
9	CH_2	24.88	1.32 (m)
			0.93 (m)
10	CH_3	9.40	0.82 (t, *J* = 7.4)
11	CH_3	12.73	0.93 (s)
12	CH_2	101.33	4.86 (t, *J* = 2.5, H_Z)
			4.78 (td, *J* = 2.0, 1.0, H_E)

[a] Carbon numbering as shown in Figure S20. [b] Chemical shifts δ in ppm; multiplicity: s = singlet, d = doublet, t = triplet, q = quartet, m = multiplet; coupling constants *J* are given in Hertz.

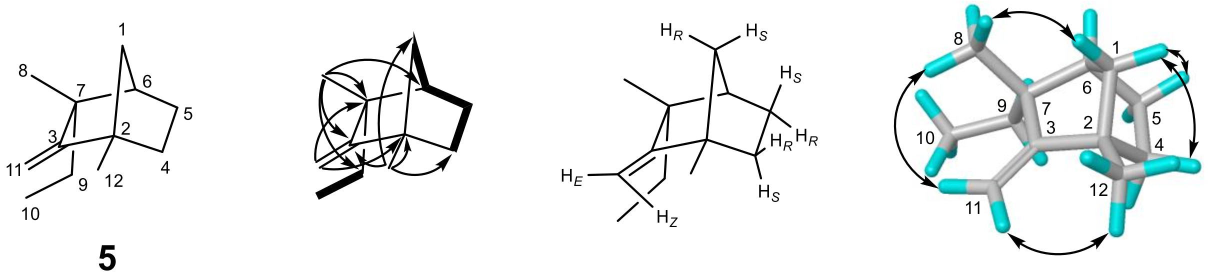

Figure S21. Structure elucidation of compound **5**. Bold: $^{1}H,^{1}H$-COSY, single headed arrows: key HMBC, and double headed arrows: NOESY correlations. Diastereotopic hydrogens are labelled H_R (*pro-R*), H_S (*pro-S*), H_E (*E*) and H_Z (*Z*).

Table S5. NMR data of **5** in C_6D_6 recorded at 298 K.

		**5**	
C[a]	type	^{13}C[b]	^{1}H[b]
1	CH_2	44.10	1.50 (m, H_R)
			1.12 (m, H_S)
2	C_q	50.09	–
3	C_q	169.66	–
4	CH_2	36.14	1.37 (m, H_R)
			1.23 (m, H_S)
5	CH_2	25.62	1.58 (m, H_S)
			1.40 (m, H_R)
6	CH	44.52	1.83 (m)
7	C_q	46.41	–
8	CH_3	25.10	0.99 (d, J = 0.7)
9	CH_2	31.93	1.49 (m)
			0.42 (m)
10	CH_3	9.48	0.84 (t, J = 7.5)
11	CH_2	97.87	4.74 (s, H_Z)
			4.62 (s, H_E)
12	CH_3	18.56	1.20 (s)

[a] Carbon numbering as shown in Figure S21. [b] Chemical shifts δ in ppm; multiplicity: s = singlet, d = doublet, t = triplet, m = multiplet; coupling constants J are given in Hertz.

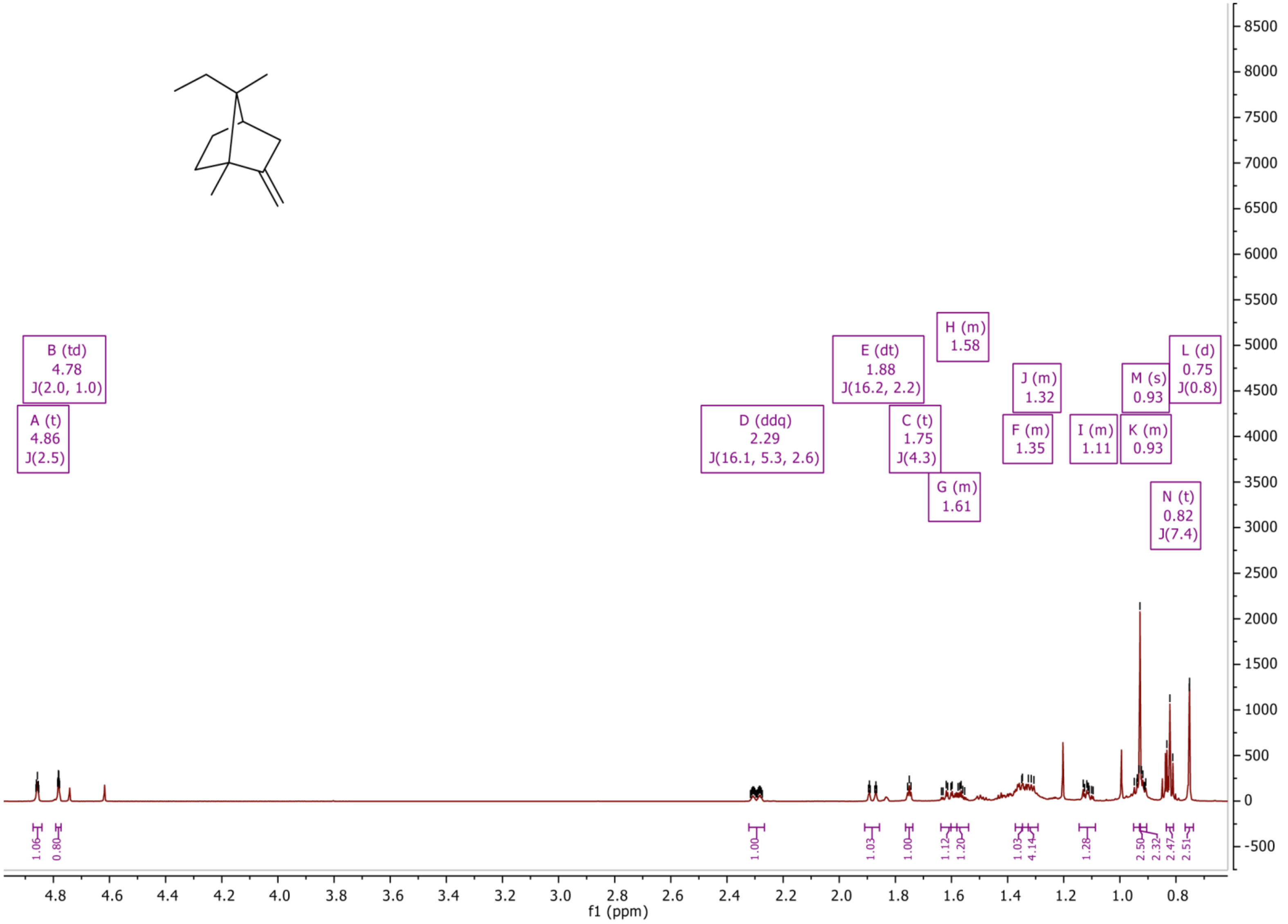

Figure S22. ^{1}H NMR spectrum of the mixture of **4** and **5** (**4**:**5** = 8:3). The signals of **4** are labelled (700 MHz, C_6D_6).

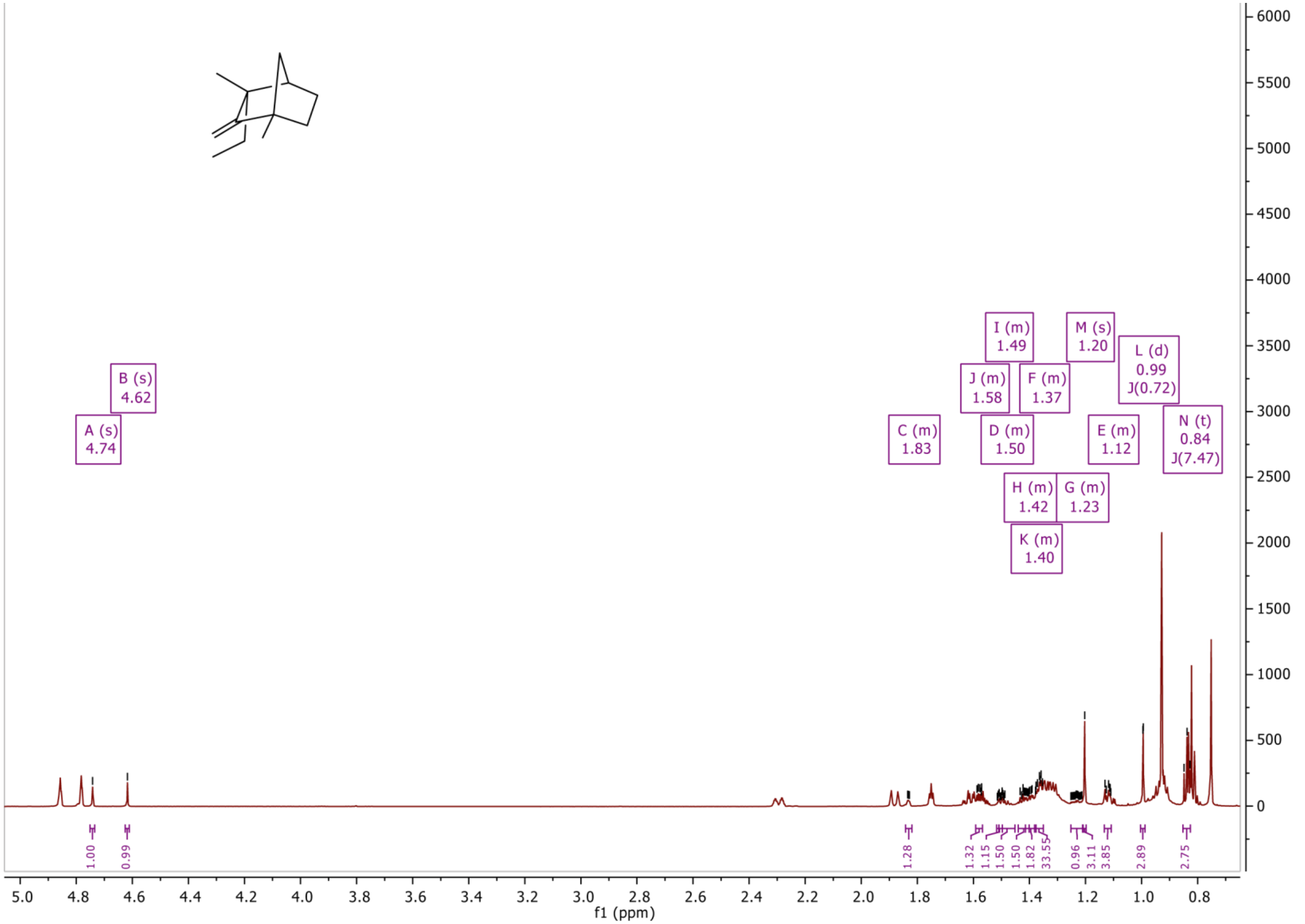

Figure S23. ^{1}H NMR spectrum of the mixture of **4** and **5** (**4**:**5** = 8:3). The signals of **5** are labelled (700 MHz, C_6D_6).

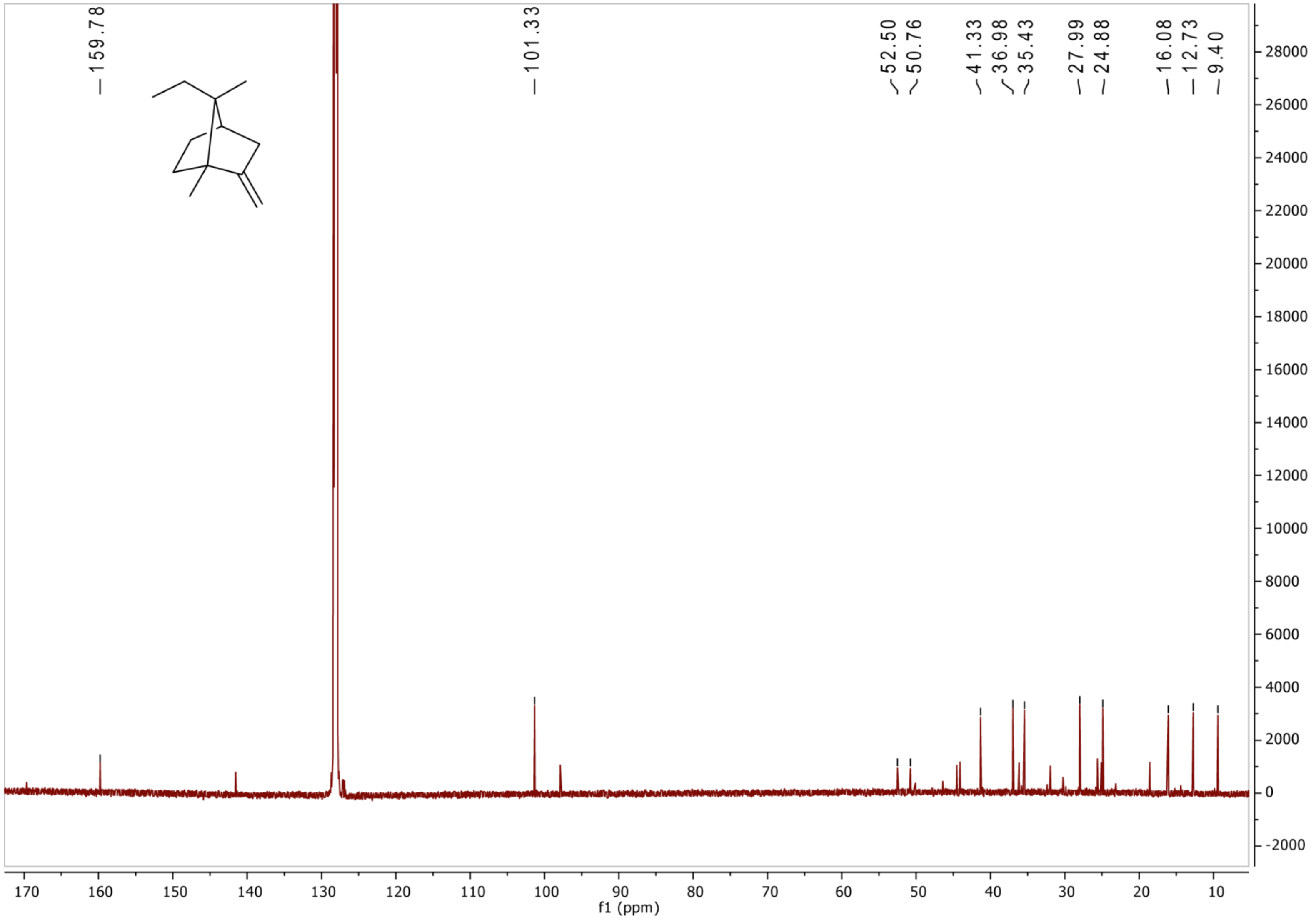

Figure S24. ^{13}C NMR spectrum of the mixture of **4** and **5** (**4**:**5** = 8:3). The signals of **4** are labelled (176 MHz, C_6D_6).

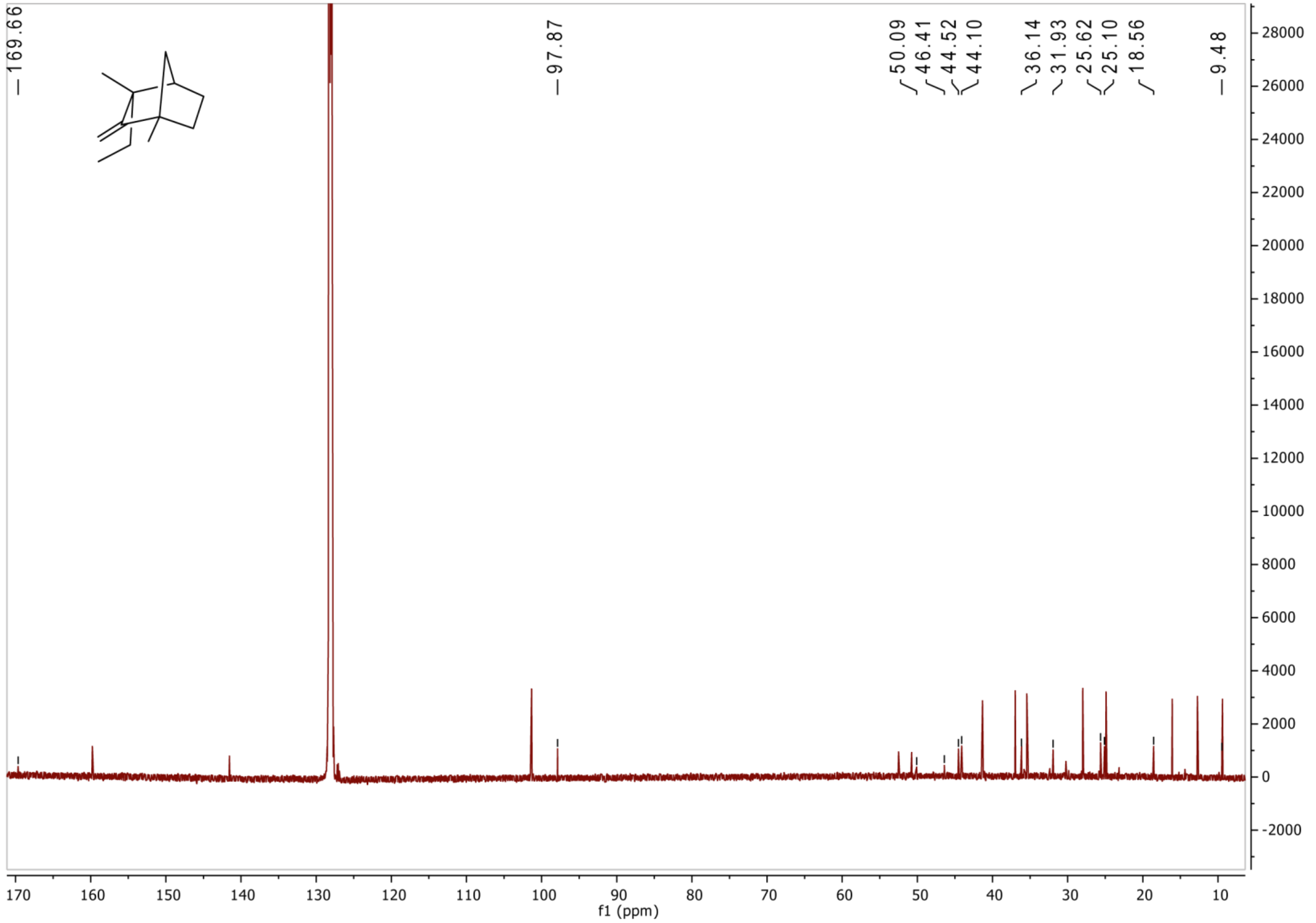

Figure S25. ^{13}C NMR spectrum of the mixture of **4** and **5** (**4**:**5** = 8:3). The signals of **5** are labelled (176 MHz, C_6D_6).

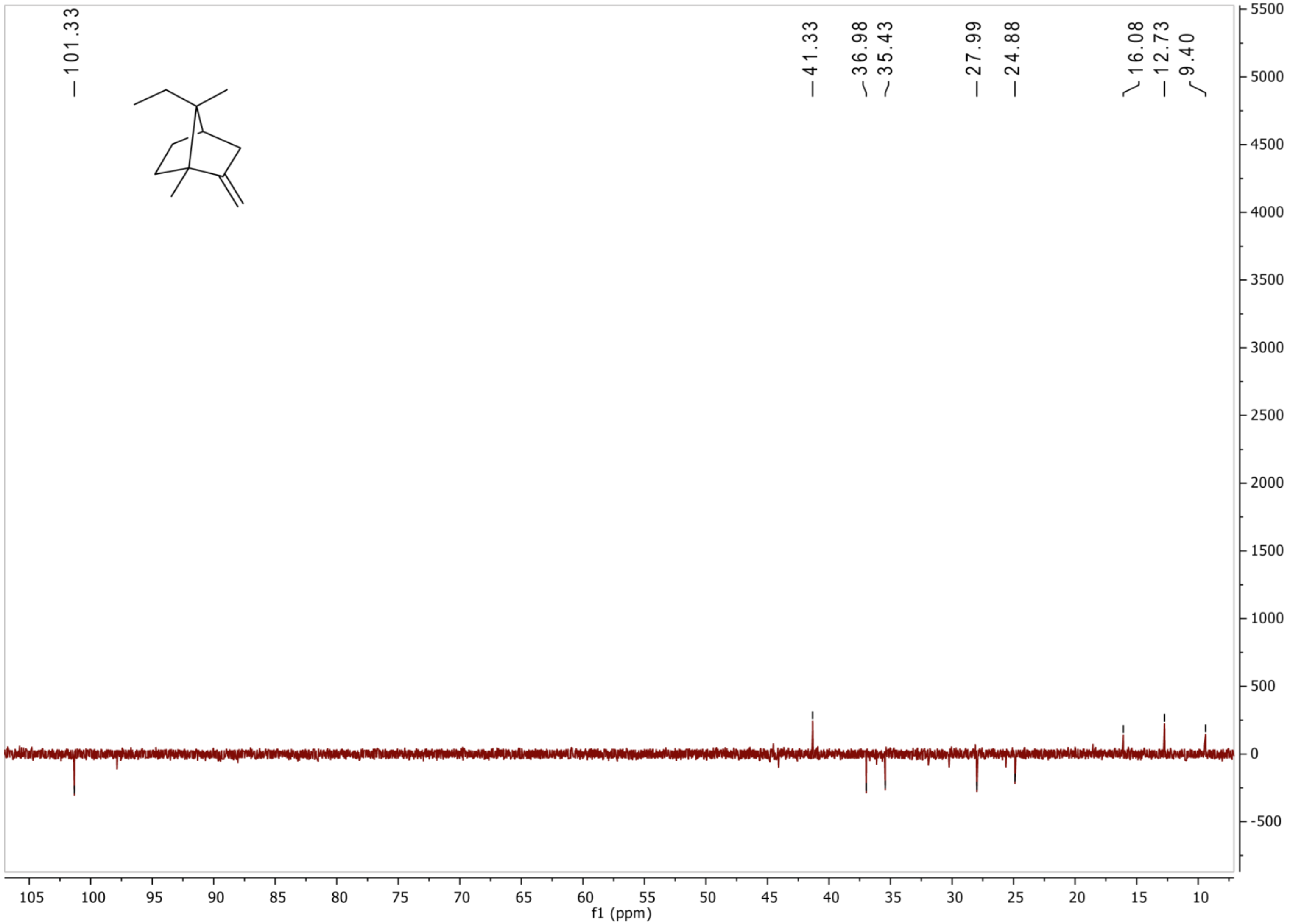

Figure S26. ^{13}C-DEPT135 spectrum of the mixture of **4** and **5** (**4**:**5** = 8:3). The signals of **4** are labelled (176 MHz, C_6D_6).

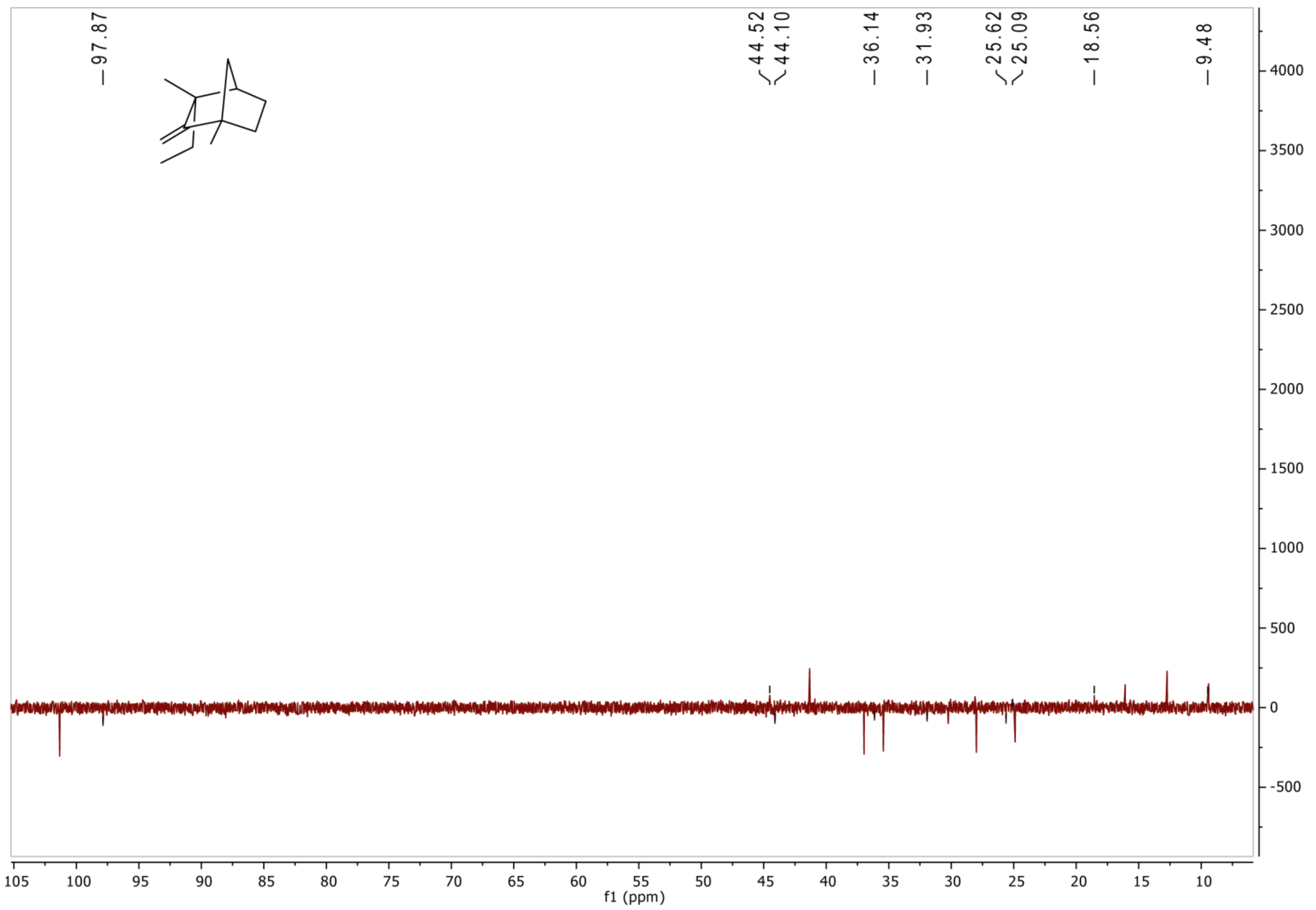

Figure S27. ^{13}C-DEPT135 spectrum of the mixture of **4** and **5** (**4**:**5** = 8:3). The signals of **5** are labelled (176 MHz, C_6D_6).

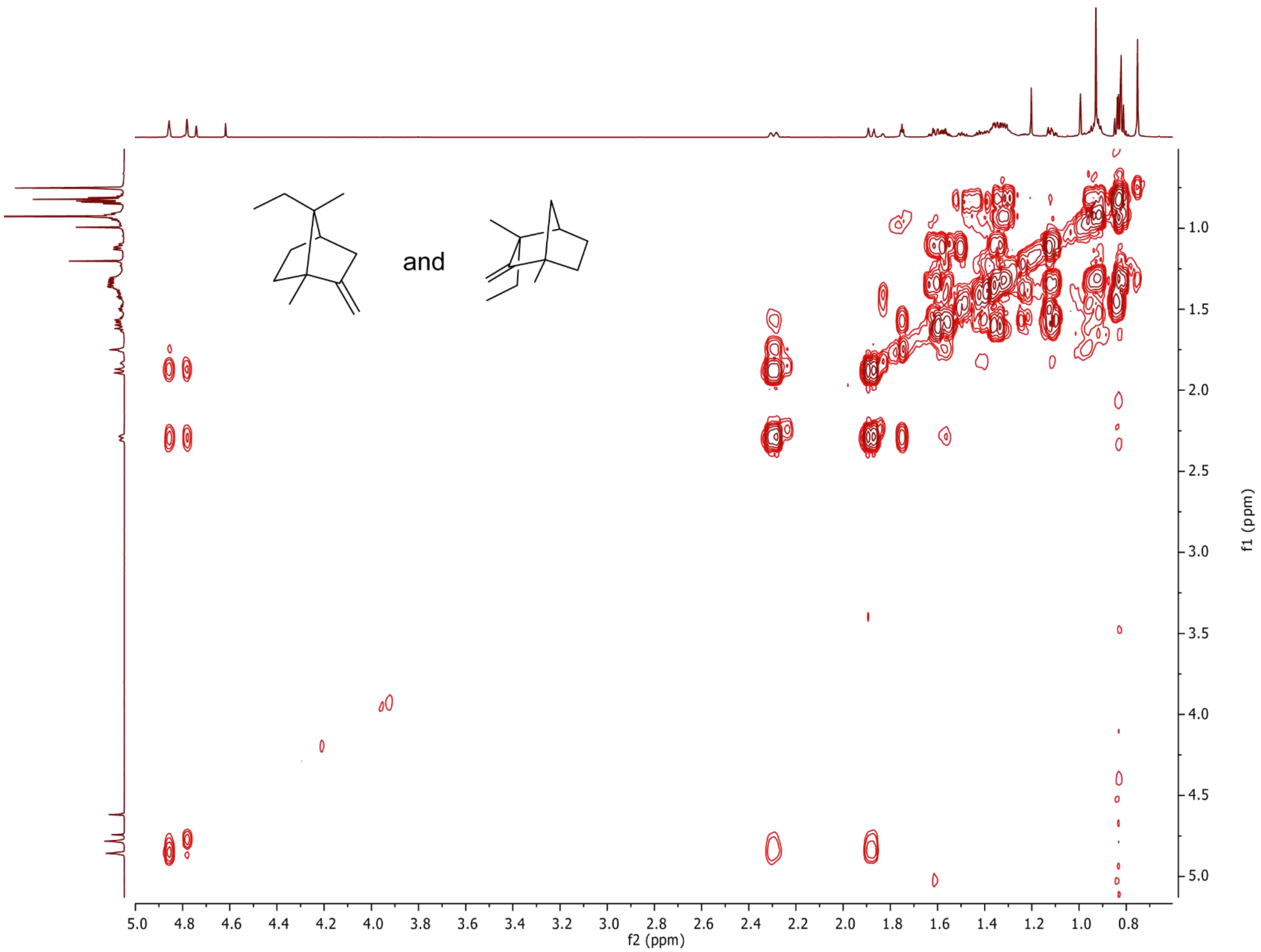

Figure S28. $^{1}H,^{1}H$-COSY spectrum (C_6D_6) of the mixture of **4** and **5** (**4**:**5** = 8:3).

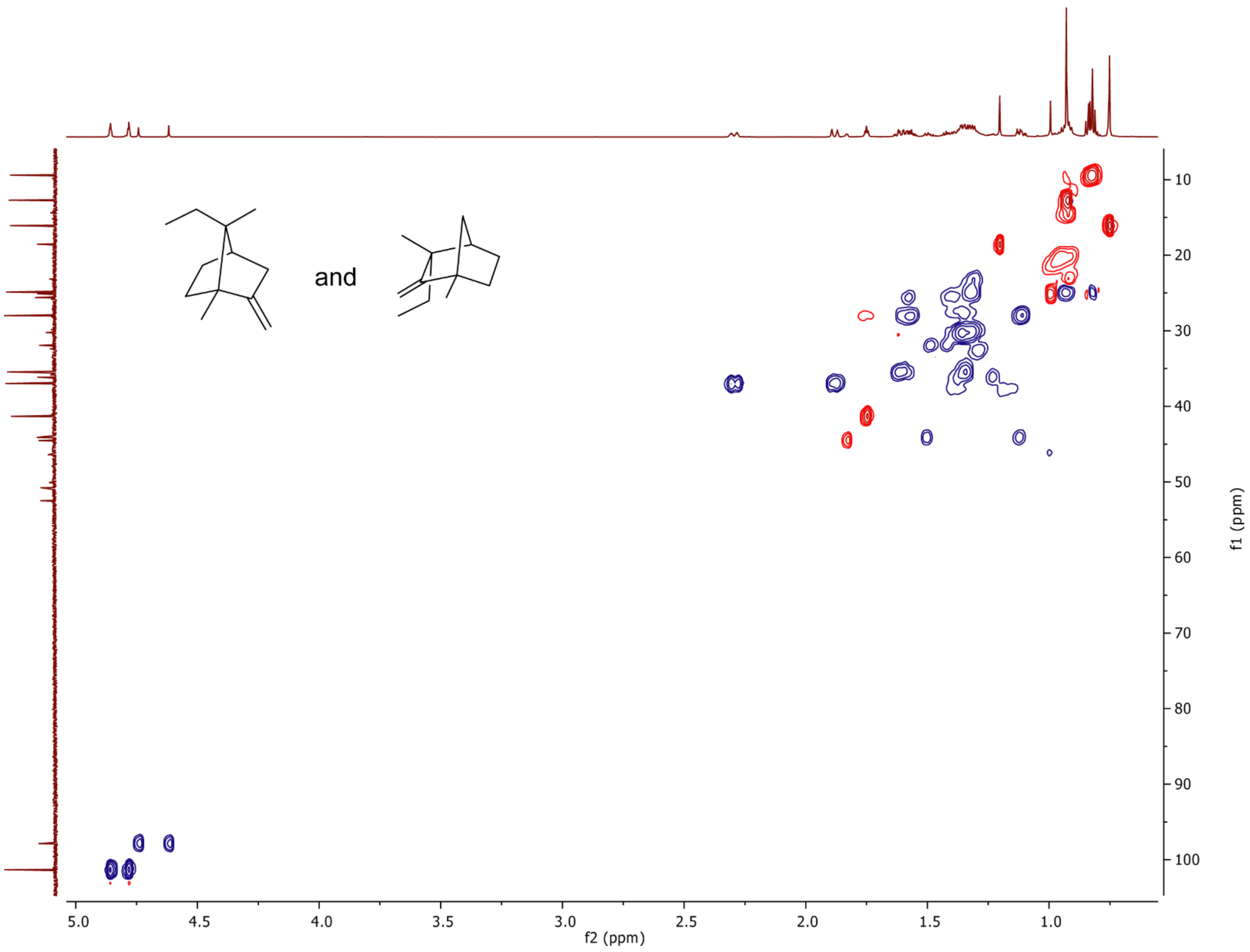

Figure S29. HSQC spectrum (C_6D_6) of the mixture of **4** and **5** (**4**:**5** = 8:3).

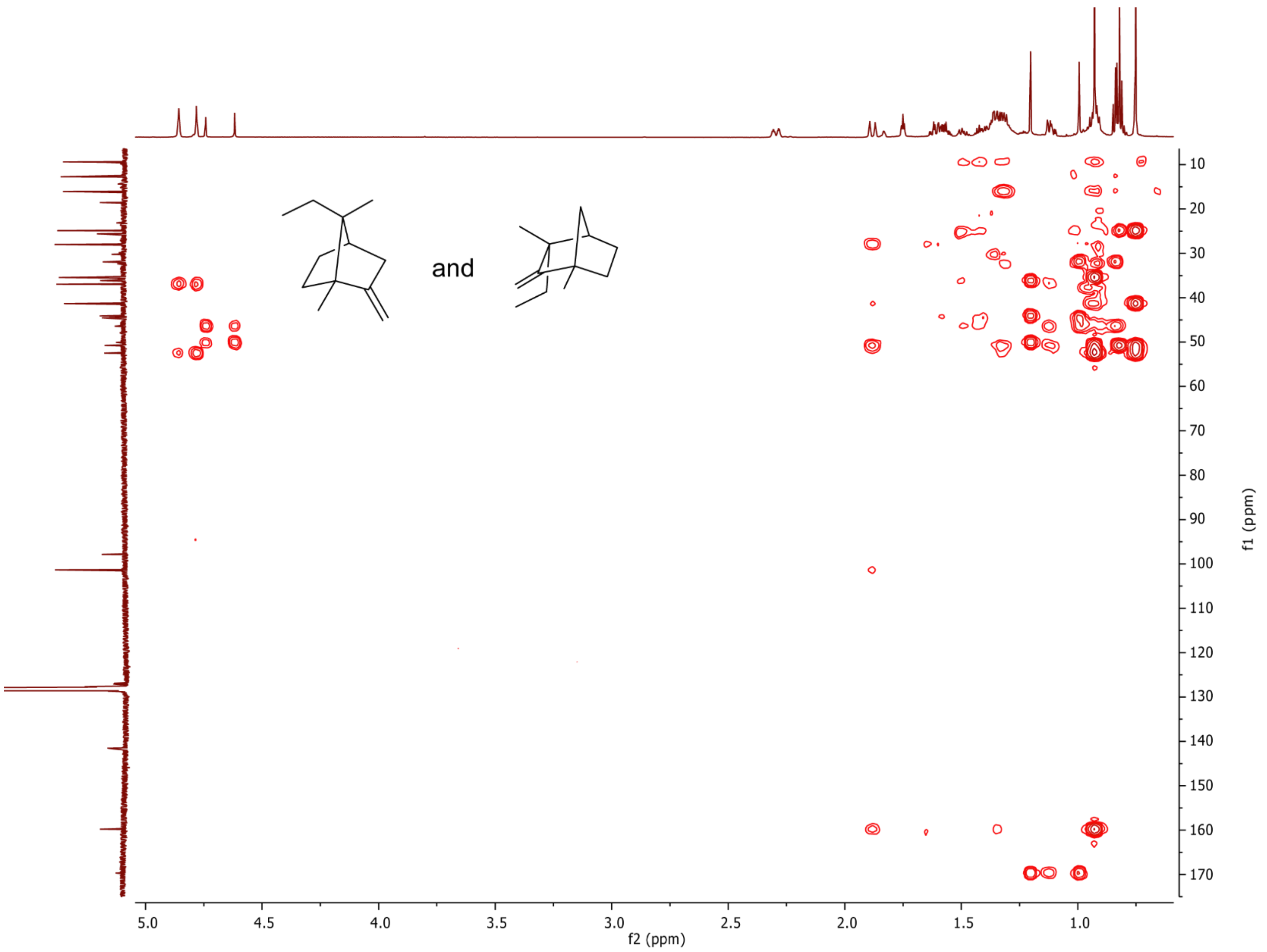

Figure S30. HMBC spectrum (C_6D_6) of the mixture of **4** and **5** (**4**:**5** = 8:3).

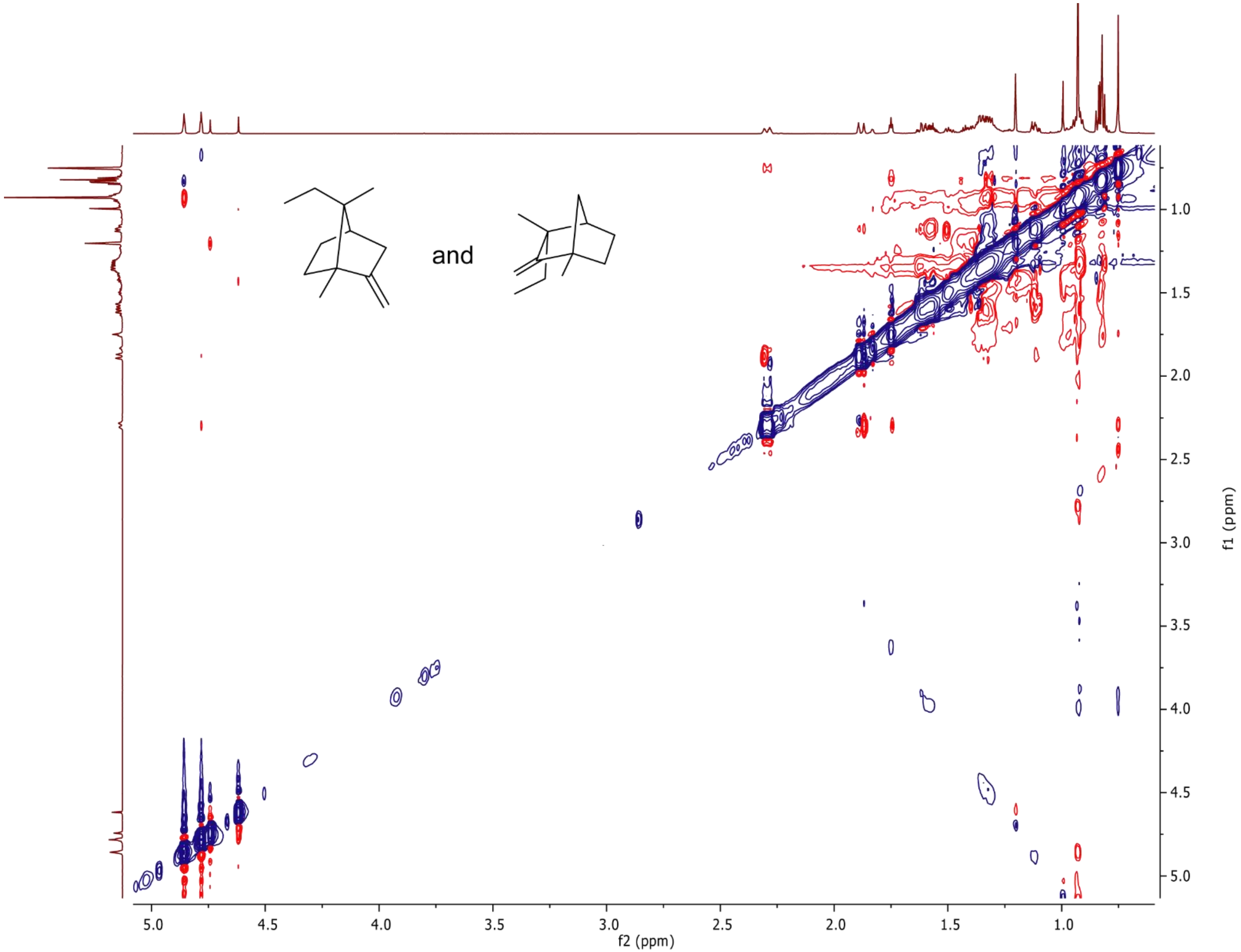

Figure S31. NOESY spectrum (C_6D_6) of the mixture of **4** and **5** (**4**:**5** = 8:3).

References

1. Gu, B.; Dickschat, J. S. *Chem. Eur. J.* **2023,** *29,* e202300775.
2. Dickschat, J. S.; Pahirulzaman, K. A. K.; Rabe, P.; Klapschinski, T. A. *ChemBioChem,* **2014,** *15,* 810–814.
3. Giets, R. D.; Schiestl, R. H. *Nat. Protoc.,* **2007,** *2,* 31–34.
4. Gu, B.; Dickschat, J. S. *Chem. Commun.,* **2022,** *58,* 4316–4319.
5. Bradford, M. M. *Anal. Biochem.,* **1976,** *72,* 248–254.
6. Gu, B.; Hou, A.; Dickschat, J. S. *Beilstein J. Org. Chem.,* **2022,** *18,* 818–824.
